# Interaction of germline variants in a family with a history of early‐onset clear cell renal cell carcinoma

**DOI:** 10.1002/mgg3.556

**Published:** 2019-01-24

**Authors:** Emmanuelle Nicolas, Elena V. Demidova, Waleed Iqbal, Ilya G. Serebriiskii, Ramilia Vlasenkova, Pooja Ghatalia, Yan Zhou, Kim Rainey, Andrea F. Forman, Roland L. Dunbrack, Erica A. Golemis, Michael J. Hall, Mary B. Daly, Sanjeevani Arora

**Affiliations:** ^1^ Molecular Therapeutics Program Fox Chase Cancer Center Philadelphia Pennsylvania; ^2^ Cancer Prevention and Control Program Fox Chase Cancer Center Philadelphia Pennsylvania; ^3^ Kazan Federal University Kazan Russia; ^4^ Department of Hematology/Oncology Fox Chase Cancer Center Philadelphia Pennsylvania; ^5^ Biostatistics and Bioinformatics Program Fox Chase Cancer Center Philadelphia Pennsylvania; ^6^ Department of Clinical Genetics Fox Chase Cancer Center Philadelphia Pennsylvania

**Keywords:** cancer risk, germline, renal cell carcinoma, succinate dehydrogenase complex, variant interaction, variants of uncertain significance

## Abstract

**Background:**

Identification of genetic factors causing predisposition to renal cell carcinoma has helped improve screening, early detection, and patient survival.

**Methods:**

We report the characterization of a proband with renal and thyroid cancers and a family history of renal and other cancers by whole‐exome sequencing (WES), coupled with WES analysis of germline DNA from additional affected and unaffected family members.

**Results:**

This work identified multiple predicted protein‐damaging variants relevant to the pattern of inherited cancer risk. Among these, the proband and an affected brother each had a heterozygous Ala45Thr variant in *SDHA*, a component of the succinate dehydrogenase (SDH) complex. SDH defects are associated with mitochondrial disorders and risk for various cancers; immunochemical analysis indicated loss of SDHB protein expression in the patient’s tumor, compatible with SDH deficiency. Integrated analysis of public databases and structural predictions indicated that the two affected individuals also had additional variants in genes including *TGFB2*, *TRAP1*, *PARP1*, and *EGF*, each potentially relevant to cancer risk alone or in conjunction with the *SDHA* variant. In addition, allelic imbalances of *PARP1 *and *TGFB2* were detected in the tumor of the proband.

**Conclusion:**

Together, these data suggest the possibility of risk associated with interaction of two or more variants.

## 1 INTRODUCTION

The annual incidence of renal cell carcinoma (RCC) in the US population was 62,700 in 2016 (Motzer, et al., 2017). The median age of diagnosis for renal cancer is 64 (Motzer, et al., 2017), with 5%–8% cases of RCC thought to be associated with an inherited genetic risk factor (Nguyen, et al., 2017). At present, most cases are diagnosed after localized invasion and spread to lymph nodes has occurred. The 5‐year survival rate for advanced disease was estimated at 11.6% for the period 2006–2012 (Motzer, et al., 2017). A better understanding of genetic factors causing predisposition to RCC would help with screening, early detection, and in improved survival. Individuals diagnosed with renal cancer at a relatively young age are more likely to have hereditary RCC, and clinical guidelines suggest referral for genetic testing for such individuals (Leung, Pan, & Shuch, 2016). Currently, genetic testing is moving from consideration of a small number of potential genes to gene panel testing and whole‐exome sequencing to establish inherited cancer‐predisposing variants. However, the enlarged data sets emerging from these tests, especially whole‐exome sequencing, can be challenging to interpret, particularly in deciphering which alterations are disease‐associated and which are not.

We present an example of integrated genomic data analysis, where identification of a rare variant by gene panel testing in the proband is extended by whole‐exome sequencing, in the proband and additional family members coupled with other clinical measurements and structural modeling of predictive novel risk‐associated variants. This work generally supports the idea that an Ala45Thr substitution in the *SDHA* (OMIM: 600857) gene is a risk factor, and implicates other potential predisposing factors for a family with a high incidence of renal cancers.

### 1.1 Clinical presentation and family history

**Figure 1:**
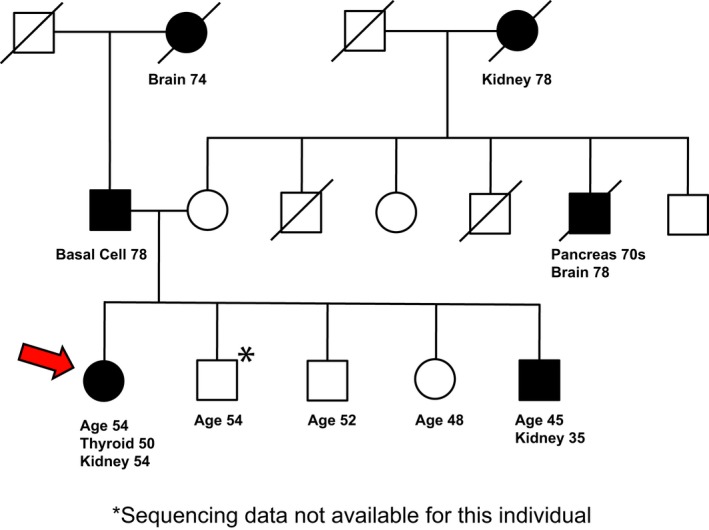
Pedigree of the female proband. The age at which the proband (designated by red arrow) and family members developed cancer as well as the types of cancers is indicated. The age at which family history was obtained is also indicated for the proband and her siblings. (*) indicated unavailable DNA. Some information has been omitted to maintain confidentiality

A woman of self‐reported German ancestry, with a personal and family history of renal cancer, was seen for genetic evaluation. At age 50, she was diagnosed with papillary thyroid cancer, which was treated with partial thyroidectomy. At 54, she was found to have a right renal mass on a surveillance CT scan. She received a partial right nephrectomy, with pathological assessment indicating stage I (pT1aN0M0), Fuhrman grade 2 clear cell renal cell carcinoma (ccRCC). Of note, the proband’s brother was found to have a large (8.5 cm) renal mass as part of a work‐up of painless hematuria that he developed at age 35. He received a radical left nephrectomy, with pathological assessment indicating a stage III (pT3N2M0), Fuhrman grade 4 ccRCC with a papillary configuration. In addition to this cancer history, the proband had a prior history of pancreatic cysts, hyperlipidemia, and uterine fibroids, while her brother had no other medical problems. Neither had a smoking history, each occasionally used alcohol and neither has ever used illicit drugs. At the time of most recent follow‐up, the proband (at 2 years after initial diagnosis of ccRCC) and her brother (9 years after diagnosis of ccRCC) did not have evidence of recurrence or metastatic disease. The proband and her brother had a strong family history of cancers, including a maternal grandmother with renal cancer (diagnosed at age 78), a maternal uncle with pancreatic and brain cancers (diagnosed in his 70s and at 78, respectively), and a paternal grandmother with brain cancer at 74 and a father with basal cell cancer at 78 (Figure 1). The proband was referred to the risk assessment clinic to establish whether inherited gene variants may contribute to this risk profile.

## 2 MATERIALS AND METHODS

### 2.1 Patient consent

All patients and their family members in the study had consented to the Fox Chase Cancer Center (FCCC) Risk Assessment Program Registry, which allowed further research genomic sequencing. Clinical information was obtained from medical records of the FCCC Risk Assessment Program. Family histories were obtained by trained genetic counselors and verified by attending physicians. Blood samples were banked in the BioSample Repository under broad consent for research and deidentified.

### 2.2 Exome sequencing

Exome sequencing of DNA was performed by BGI Americas Corporation (Cambridge, MA, USA) at 100× average coverage. Agilent SureSelect XT All Exon V6 kit was used for exon capture (Agilent Technologies, Wilmington, DE, USA). Library preparations were done using Illumina standard protocol. Each captured library was indexed, then loaded onto Hiseq2000 platform (Illumina, Hayward, CA, USA) for 100 bp paired‐end high‐throughput sequencing. Sequence reads were mapped to human reference genome (hg19) using the Burrows‐Wheeler Aligner (BWA) (Li & Durbin, 2009). Single Nucleotide Polymorphisms (SNPs) and small Insertion/Deletions (InDels) were detected using Genome Analysis Toolkit (GATK) (McKenna, et al., 2010).

### 2.3 Variant annotation

Variant annotations were computed with ANNOVAR (Wang, Li, & Hakonarson, 2010) version 2017‐07‐16 and include: (1) population frequency, from (a) ANNOVAR‐provided versions of the gnomAD genome and exome call sets (Lek, et al., 2016), version 2017‐03‐11 release (b) ExAC (Lek, et al., 2016),as provided in the ANNOVAR database version 2015‐11‐29; (2) predicted protein impact, using ANNOVAR‐provided versions of RefSeq (O'Leary, et al., 2016) (3) predicted deleteriousness, using the ANNOVAR file dbnsfp30a (Liu, Wu, Li, & Boerwinkle, 2016).Annotations from InterVar (Li & Wang, 2017), a bioinformatics software tool for clinical interpretation of genetic variants by the ACMG/AMP 2015 guidelines (Richards, et al., 2015) were also included in the report. In addition, variants detected in the proband and her siblings were annotated with parent of origin, computed using the genomes of the parents.

### 2.4 Variant filtering and prioritization

Selected annotations were applied to filter and prioritize the variants prior to manual evaluation. (a) The first step was a hard‐filtering step intended to exclude variants highly unlikely to be causative. Variants were required to have genotype quality ≥10, read depth ≥10, and maximum population frequency <0.01. (b) Variants were also required to be predicted to impact the protein sequence derived from any RefSeq transcript, or to be previously reported as pathogenic or likely pathogenic by at least one of the variant effect prediction tools (SIFT, POLYPHEN2, LRT, MutationTaster, MutationAssessor, or CADD) or need to be located in the splicing site (2 bp from exon/intron boundary). (c) Variants were also tested by BGI for segregation with the observed phenotypes in the family. Segregation was tested for de novo, autosomal dominant, homozygous recessive, compound heterozygous, X‐linked, and imprinted inheritance patterns. Incomplete penetrance was modeled by retaining non‐segregating variants in genes with phenotype matches to the proband’s features. For a candidate gene approach, datasets of renal cancer‐related genes, obtained from multiple sources, both commercial and publicly available were integrated. Renal cancer‐specific genes were obtained from Ingenuity, TCGA (using cBioPortal), ICGC, and HGMD Professional databases; genes implicated in multiple cancers (cancer census genes) were obtained from COSMIC, database. Subsequently, our candidates were prioritized by building a network using Cytoscape program and MiMi plugin, and subsequently only retaining the genes that were either nominated by two or more sources, or nominated by one database but interacting with than two or more high‐confidence genes. The list is available on request. GenBank reference sequence and version number for all the gene(s) that were studied is as follows, *SDHA—* NC_000005.10, *MEN1*—NC_000011.10, *RECQL4*—NC_000008.11, *PARP1*—NC_000001.11, *EGF*—NC_000004.12, *TRAP1*—NC_000016.10, *TRIB3*—NC_000020.11, *TGFB2*—NC_000001.11.

### 2.5 DNA extraction from FFPE

DNA extraction from FFPE was performed by following standard protocols (Senguven, Baris, Oygur, & Berktas, 2014). Briefly, tumor cells were collected from an H&E stained section that was demarcated by a pathologist. The cells were deparaffinized by incubation with xylene at 56°C for 1 hr. Xylene was removed and the cells were washed with descending concentrations of ethanol and the pellet was allowed to dry at 56°C for 10 min. This was followed by DNA isolation using the QIAmp DNA micro kit (Qiagen catalog number 56304). The isolated DNA was measured on Nanodrop, and used for further experiments.

### 2.6 Sanger sequencing

The sequences of the primers used to validate the exome sequencing data are listed above the chromatograms in the Supporting Information Appendix S1. The sequences to test the tumor DNA for *SDHA* LOH by deletion were CAGTTTGCAAGGGGAAATTACT and AGCATGAACTTACGGAATCTGA. The sequences of the primers used to check for allelic imbalance in chromosome 1q in the tumor were ATTCACCATTGAGGGCATAGG and ACATAAGCACAGCTCAGAAGG for ADAMTS4 rs41270041, TAGAGGTGACATAGGGACACA and CATACGTCTCATTTGCTGCTTC for ATF6 rs2070151, AGTAGAGTCCAGAGAGGTTACG and GAGCTGAGAATCTTCTGATGGG for PARP1 rs3219143, CATCCATCTGCCTCTCATCTTC and GCCTTTGTTTCCTCTCTGTCT for DISC1 rs821616, GAGGAATCGTTGGCATCCTT and CTAACCGTGCTGGCCTATG for OPN3 rs2273712. The PCR samples were sent to Genewiz (Plainfield, NJ, USA) for Sanger sequencing.

### 2.7 Analysis of variants reported in TCGA and other studies

CBioPortal (Cerami, et al., 2012, http://www.cbioportal.org) was used to access data from the most recent TCGA studies based on frequency of somatic alterations (amplification, mutation, deletion) in *SDHx *genes (data downloaded on 11/22/2017). The names of the multiple studies downloaded from http://www.cbioportal.org are as follows: Clear Cell Renal Cell Carcinoma (U Tokyo, Nat Genet 2013), Kidney Renal Clear Cell Carcinoma (BGI, Nat Genet 2012), Kidney Renal Clear Cell Carcinoma (TCGA, Provisional), Multiregion Sequencing of Clear Cell Renal Cell Carcinoma (IRC, Nat Genet 2014), Kidney Chromophobe (TCGA, Provisional), Kidney Renal Papillary Cell Carcinoma (TCGA, Provisional), Renal Non‐Clear Cell Carcinoma (Genentech, Nat Genet 2014), Targeted gene sequencing in 62 high‐grade primary Unclassified Renal Cell Carcinoma (MSK, Nature 2016), GENIE Renal Clear cell, GENIE Papillary Renal Cell Carcinoma, GENIE Chromophobe. We also analyzed frequency of somatic mutations (corrected by gene length) in *SDHA*, *SDHB*, *SDHC,* and *SDHD* in all renal cancers by combining data from TCGA (http://www.cbioportal.org/), COSMIC (Forbes, et al., 2017) (http://cancer.sanger.ac.uk/cosmic) and International Cancer Gene Consortium (Zhang, et al., 2011) (ICGC, http://icgc.org/; see Supporting Information Figure S2, Supporting Information Data S5). The somatic missense variants reported in the relevant TCGA studies were analyzed using Annovar prediction tools, including SIFT, PolyPhen 2.0, MutationAssessor (Wang, et al., 2010; see Supporting Information Data S5). Those predicted to be damaging by a consensus of at least four out of nine protein function prediction programs were mapped to a schematic of the SDHA protein. Similarly, germline variants (missense and nonsense) that have been reported as pathogenic or likely pathogenic in the ClinVar database (https://www.ncbi.nlm.nih.gov/clinvar/ ) were mapped to a schematic of the SDHA protein (data obtained on 9/18/2017; Landrum, et al., 2016).

### 2.8 Immunohistochemistry

Standard immunohistochemical (IHC) staining was performed on FFPE whole‐tissue section. The sections were rehydrated and antigen was retrieved using citrate buffer at pH 6. IHC was performed using antibodies for SDHA (cat no. ab14715; Abcam, Cambridge, MA, USA, used at 1:1,000 dilution) and SDHB (cat no. 14714; Abcam, used at 1:400).

### 2.9 Modeling TRAP1/substrate interactions

There are several experimental structures of human (Lee, et al., 2015; Park, et al., 2017) and *Danio rerio* TRAP1 (Elnatan, et al., 2017; Lavery, et al., 2014) in various functional states. The human structures consist of residues 60–561 while the zebrafish structures contain the complete C‐terminal domain of TRAP1, equivalent to residues 60–704 of the human sequence. The structure of full‐length human TRAP1 (excluding the transit peptide, residues 1–59) was modeled with Biological Assembly Modeler (Shapovalov, Wang, Xu, Andrake, & Dunbrack, 2014). In the absence of a structure of TRAP1 in complex with a client protein, a cryo‐EM structure of human HSP90 in complex with CDK4 (PDB 5FWM) was used to model the interaction chaperone‐client. In this structure, the N and C‐terminal domains of CDK4 are separated from each other by 35 Å. The intervening sequence, consisting of the last strand of the *N*‐terminal domain beta sheet and the hinge region, is threaded between the two protomers of the HSP90 homodimer. A model of how TRAP1 may interact with substrates by superimposing the middle domain of one HSP90 monomer in the cryo‐EM structure onto the middle domain of one monomer of *Caenorhabditis elegans* TRAP1 (PDB 4IYN) was generated (Lee, et al., 2015). The folded domains of CDK4 were removed for depiction of the resulting model. All molecular display figures were prepared with PyMOL.

## 3 RESULTS

### 3.1 Genomic analyses

DNA from the proband was first assessed with a commercial ~80 gene multi‐cancer panel with genes associated with hereditary cancers across eight major organ systems, including the genitourinary tract. This revealed no defined pathogenic variant, but two variants of uncertain significance (VUS): c.133G>A (p. Ala45Thr) in *SDHA*, which encodes a subunit of the succinate dehydrogenase complex, and c.308C>T (p. Pro103Leu) in *RECQL4 *(OMIM: 603780)*,* which encodes a RecQ family helicase, were identified. Genetic test results of the patient’s brother revealed the same variants in *SDHA* and *RECQL4*, and an additional VUS predicted to affect protein sequence: c.1098A>T (p. Glu366Asp) in *MEN1 *(OMIM: 131100), encoding the menin tumor suppressor associated with multiple endocrine neoplasia. As these results did not definitively assign a known genetic risk factor, whole‐exome analysis was performed on peripheral blood DNA of the female proband (at age 54), her parents, her affected brother (at age 45), and two unaffected siblings (ages 46 and 52). Germline DNA from other family members and tumor DNA from the affected brother were not available.

To analyze this data, we integrated several filtering approaches (detailed in Methods) to select variants of interest, in the context of their frequency in the general population, and prior knowledge associating specific genes or variants with hereditary cancers or sporadic RCC (e.g., genes with roles in DNA repair, or functionally related to known oncogenes). Variants changing protein coding sequences or in splice sites that passed this filtering that were identified in the proband or her affected brother were further analyzed for family distribution. A separate analysis of unbiased family segregation analysis was performed by BGI. Tools in Annovar and InterVar predicted damaging consequences of detected variants. We note, although the proband self‐reported as of German ancestry, genomic analysis indicated some African ancestry (see Supporting Information Appendix S1).

Table 1 shows some characteristics of high‐quality variants identified by these criteria. The full Annovar‐InterVar report and the quality of the reads for all individuals are provided in Supporting Information Data S1. All the variants identified by exome sequencing were confirmed by Sanger sequencing (Supporting Information Data S2). Upon analysis of distribution in family members for whom sequence information was available, the variant in *RECQL4* identified by initial panel analysis was noted only as potentially damaging (and was identified in two unaffected siblings), and the *EGF *(OMIM: 131550), variant was only identified as damaging by one prediction program; both were inherited through the father. The *MEN1* variant, also inherited through the father, was predicted to be damaging, but was not found in the proband, and was found in the two unaffected siblings, suggesting less relevance. The other variants identified in the affected individuals, including missense variants in *SDHA*, *TRAP1*, (OMIM: 606219), *PARP1 *(OMIM: 173870), *EGF*, and *TRIB3 *(OMIM: 607898), were predicted to be potentially or likely function damaging. In addition, a compound heterozygosity was found in *TGFB2*. Based on family segregation analysis, the interaction between the *SDHA*, *TRAP1*, *TGFB2*, and potentially *EGF *variants was considered of interest (Table 1). Among the variants, the greatest interest was placed in *SDHA*, given studies implicating this gene in renal cancer pathogenesis.

**Table 1:** Selected variants with identification in dbSNP, distribution in the family, predictor scores and frequencies in GnomAD total population for all variants and in African population for the variant of maternal origin

Gene	Variant	dbSNP	Mr.	Fr.	Pro.	Br.	Sr.	Br.	Damage pred.	GnomADAll	GnomADAfrican
*SDHA*	Missense NM_004168 c.G133A:pA45T	rs140736646							3	113/276942 (0.00040)	2/24038 (0.000083)
*MEN1*	Missense NM_000244 c.C1113A:pE371D	rs149383809							6	19/246256 (0.000077)	
*RECQL4*	Missense NM_004260 c.C308T:pP103L	rs199543866							P	164/275542 (0.00060)	
*PARP1*	Missense NM_001618 c.A370G:pT124A	rs139924814							3	4/277196 (0.000014)	
*EGF*	Missense NM_001963 c.G47C:pS16T	rs200394315							1	69/276870 (0.00025)	
*TRAP1*	Missense NM_001272049 c.C1640G:pT535S	rs77440336							4	723/277212 (0.0026)	643/24032 (0.027)
*TRIB3*	Nonsense NM_021158 c.C106T:pR36X	rs150841542							—	26/277142 (0.000094)	25/24028 (0.0010)
*TGFB2*	5′UTR NM_003238 c.−52delT	rs200186989							—	99/30916 (0.0032)	
*TGFB2*	5′UTR NM_003238 c.−45_44insA	rs758747010							—	1/199016 (0.0000050)	0/13324 (0)

Notes

In the damage prediction column, the numbers represent the number of Annovar prediction tools that annotated the variant as damaging, deleterious or to cause loss of functionality; P means a prediction of possible damaging. In the GnomAD columns, the numbers represent the allele count over the total number of alleles reported. The value of the ratio is under parenthesis. A table providing total reads, total mapped reads, coverage at target region, is presented in Supporting Information Data S7.

GenBank reference sequence and version number of the gene(s) studied: *SDHA—* NC_000005.10, *MEN1*—NC_000011.10, *RECQL4*—NC_000008.11, *PARP1*—NC_000001.11, *EGF*—NC_000004.12, *TRAP1*—NC_000016.10, *TRIB3*—NC_000020.11, *TGFB2*—NC_000001.11.

### 3.2 SDHA Ala45Thr variant

*SDH* genes, commonly collectively referred as *SDHx*, function as tumor suppressor genes in hereditary paragangliomas, pheochromocytomas, and gastrointestinal stromal tumors (GISTs) (Renkema, et al., 2015). SDH‐deficient renal carcinomas were first identified in 2004 (Vanharanta, et al., 2004) and accepted as a unique subtype of renal tumor in 2016 (Moch, Cubilla, Humphrey, Reuter, & Ulbright, 2016). Two recent studies have identified the variant SDHA p.Arg31stop, previously defined as the most prevalent mutation observed in SDH‐deficient (GISTs) (Miettinen & Lasota, 2014), in renal cancer patients (Carlo, et al., 2018; McEvoy, et al., 2018). Association of *SDHx* germline variants with thyroid cancer risk has also been described (Neumann, et al., 2004; Ni, et al., 2012), of particular relevance to the proband in this study.

The SDHA Ala45Thr (rs140736646) variant is most prevalent in Europeans, with the GnomAD database (Lek, et al., 2016) indicating presence in one in 651 individuals. With this representation in the general population, the variant is not a credible candidate to be considered disease‐causing in a monogenic disorder. However, this representation level is compatible with a role as a low penetrance risk modifier, which can be seen at common, intermediate, or rare frequencies (Kousi & Katsanis, 2015). Considering that epidemiological studies have shown that a family history of renal cancer strongly extends the risk to first‐, second‐, and third‐degree relatives (Clague, et al., 2009; Gago‐Dominguez, Yuan, Castelao, Ross, & Yu, 2001; Gudbjartsson, et al., 2002; Schlehofer, et al., 1996), the finding that the allele was inherited from a mother whose mother had also been affected by kidney cancer (Figure 1) added to the interest of this VUS (see also Supporting Information Data S3).

**Figure 2:**
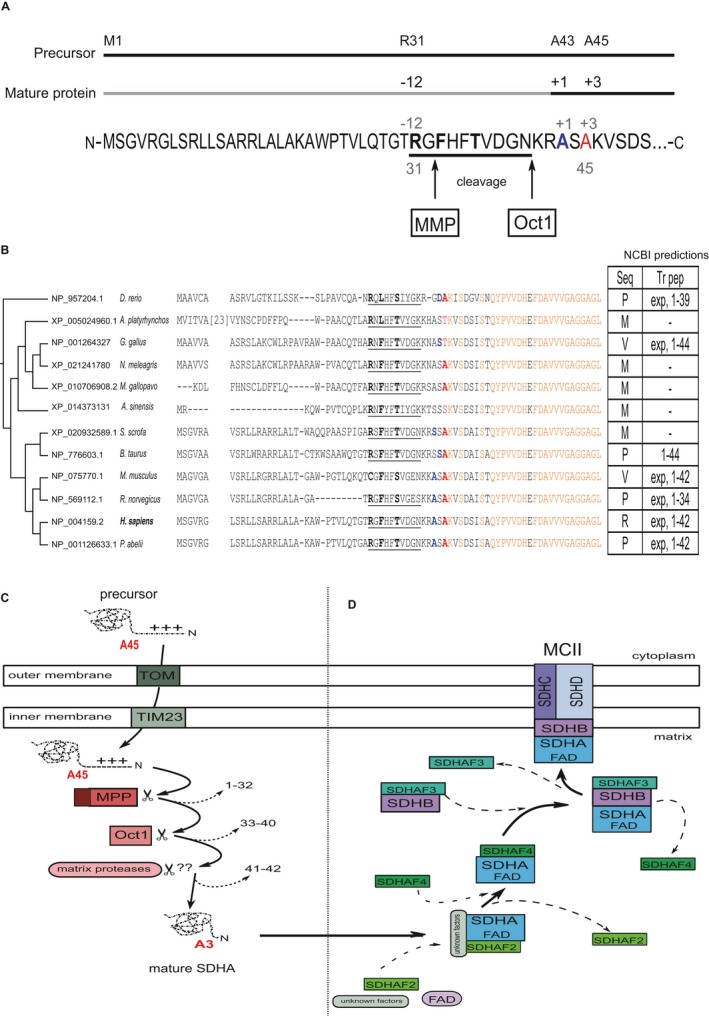
SDHA sequence analysis and SDHA assembly into an SDH complex. (a) *N*‐terminal schematic and sequence of precursor and mature human SDHA. The presequence with octapeptide motif and its cleavage sites by the mitochondrial processing peptidase (MMP) and the mitochondrial intermediate peptidase (MIP) octapeptidyl aminopeptidase 1 (Oct1) are indicated. Cleavage results in a mature protein with a neo‐terminus ASAKVS (initial A in blue font); the alanine variant in the proband corresponds to position 3 (red font). R31 indicates the position in the presequence of an amino acid often mutated to stop codon in PGL and GIST (Casey, et al., 2017). The motifs that function in a cell to target proteins to their final destinations are short stretches defined by a consensus sequence with some relatively fixed and some flexible amino acids (Kohda, 2017). The more important fixed residues are promiscuously recognized by various proteins including a translocase and a peptidase if the signal is removed from the precursor. The role of the others is not so clear. (b) The signal sequence has a characteristic RX(↓)(F/L/I)XX(T/S/G)XXXX(↓) motif at residues ~31–40. Sequence alignment in various organisms showing low conservation of the presequence (Calvo, et al., 2017) and high conservation of mature peptide sequence (in orange). Ala45 (in bold red) is highly conserved among the mammalian species. In cases where the first amino acid of the mature sequence has been confirmed it is indicated by bold blue font. The presence of a serine or an alanine at the *N*‐terminus is typical for mitochondrial proteins and consistent with the *N*‐rule in bacteria describing that stabilizing amino acids are typically found at the *N*‐termini of mature proteins (Tasaki, Sriram, Park, & Kwon, 2012; Varshavsky, 1997). The NCBI RefSeq database status of the sequences and the cleavage prediction are indicated in the table (right), where Seq indicates NCBI RefSeq sequence status, and Tr pep indicates NCBI prediction for transit peptide. P: provisional, M: model, V: validated, R: reviewed, Exp: experimentally validated. Numbers represent the transit peptide amino acids. The mammalian sequences for SDHA cluster separately from the chicken, duck, and other variants of this protein in lower vertebrates, largely because of differences involving the presequence. The presence of a the variant amino acid (Thr) in chicken (*Gallus gallus*) and duck (*Anas platyrhynchos*) is not considered as a reason to dismiss the variant, as examples in which a disease‐causing variant correspond to the wild‐type allele in another species have been reported (Azevedo, et al., 2016). Further discussion on the processing of the presequence and of the potential role of Ala45 is presented in Supporting Information Appendix S1. In chicken and in cow, the first residue of the mature protein aligns with the second residue in the other species. (c) Schematic representation of the mitochondrial presequence import pathway. The SDHA precursor is translocated through the outer and inner mitochondrial membranes by the Translocator of the Outer Membrane (TOM) and Translocator of the Inner Membrane (TIM) complexes, followed by the sequential proteolytic cleavages described in (a). Arrows indicate successive cleavages by MMP and Oct1, previously identified in yeast Sdh1 (Branda & Isaya, 1995). (d) Step‐wise assembly of SDH complex (also known as Mitochondrial Complex II, MCII). After flavination (addition of FAD) of mature SDHA, mediated by SDHAF2, SDHAF4 binds to SDHA to reduce auto‐oxidation. SDHAF3 facilitates the formation of an SDHA‐SDHB complex that assembles with SDHC and SDHD located in the inner membrane

*SDHA* encodes the flavoprotein (Fp) subunit of the succinate dehydrogenase (SDH) complex (composed of SDHA, SDHB, SDHC, and SDHD), which forms part of complex II of the mitochondrial electron transport chain, and is responsible for transferring electrons from succinate to ubiquinone (coenzyme Q). As for most nuclear‐encoded mitochondrial matrix proteins, SDHA is synthesized with a presequence and matures through the presequence cleavage pathway (Figure 2a,b). Removal of this presequence (Figure 2c) is required for the FAD attachment to SDHA (Robinson & Lemire, 1996), which occurs before SDH complex assembly (Figure 2d; see further discussion on the interaction of SDHA with the mitochondrial import pathway in Supporting Information Appendix S1). Ala45 in the preprotein becomes Ala 3 in the mature protein. We compared the presequence and adjacent *N*‐terminal sequences from the mature protein across multiple vertebrates. Beginning with residue Ser44, the mature protein is evolutionarily strongly conserved; further, all substitutions involving the Ala45 position in mammals occur in non‐mammalian species, suggesting a potentially important conserved role for this residue.

SDHA immunochemistry on FFPE sections has been validated for the identification of patients with pathogenic germline variants in *SDHA* (Korpershoek, et al., 2011). SDHA‐deficient tumors lose protein expression of both SDHA and SDHB (Papathomas, et al., 2015), as direct interaction between these two proteins within the larger SDH complex is essential for protein stability. To evaluate whether the Ala45Thr variant was associated with changes in expression of these proteins, we used immunohistochemistry (IHC) to assess SDHA and SDHB protein levels in normal renal and RCC tumor tissue from the female proband. An SDHB‐negative gastrointestinal stromal tumor (GIST) from a patient with a heretozygous germline *SDHA* 3‐base deletion that spans the IVS4/exon 5 junction (c.457‐2_c457delAGC) (Belinsky, et al., 2013) and two RCC tumors with clear cell features from a patient with wild‐type *SDHA* and *SDHB* were used as controls.

**Figure 3:**
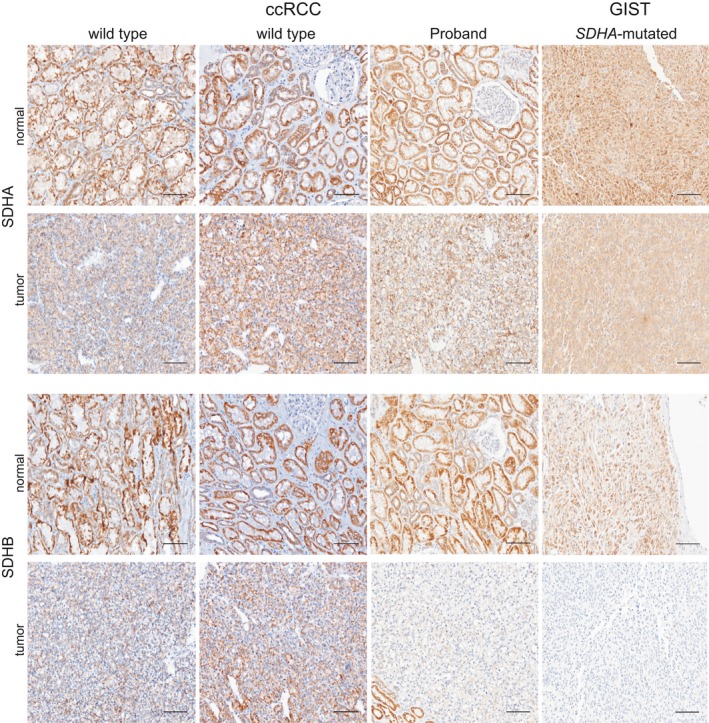
SDHA and SDHB expression in the proband versus control tissue. Shown, SDHA and SDHB expression visualized by IHC in the normal renal tissue and ccRCC from two patients with wild‐type *SDHA* (panel 1 and 2, positive control) and from the proband (panel 3). Shown in panel 4 is normal gastrointestinal tissue and GIST tumor from a patient with confirmed S*DHA*‐inactivating mutation (splice site mutation: IVS 4‐exon 5) which causes SDHB loss (negative control). The proband panel shows reduced SDHB expression in the tumor compared to the positive and negative controls (for additional internal IHC controls, see Supporting Infomation Figure S1). Magnification: 20×. Scale bar: 100 µm

The expression of SDHA was unaffected in tissue from the proband (Figure 3). Strikingly, the expression of SDHB was unaffected in normal renal tissue from the proband, but strongly reduced in neoplastic tissue from the proband (Figure 3). To exclude a possible false‐negative signal for SDHB staining in ccRCC (Cornejo, et al., 2015; Gill, 2018), we also analyzed the endothelial cells of blood vessels within the tumor and normal renal epithelium as a positive control (Supporting Infoamtion Figure S1a,b). At the junction between tumor and normal renal tissue for the proband (S1A), SDHB staining is seen in normal renal epithelium and in endothelial cells, but not in tumor cells. For comparison, SDHB staining in endothelial cells from the *SDHA *wild‐type ccRCC tumor and *SDHA*‐mutated GIST tumor is shown in S1C and S1D, respectively. Interestingly, while the loss of SDHB protein expression detected by IHC is associated with pathogenic *SDHA* variants, the tumor from the proband had the characteristics of a clear cell carcinoma without additional features typically associated with SDH‐deficient RCC, such as eosinophilic cytoplasm with intracytoplasmic vacuolations and inclusions (Gill, et al., 2014, 2011; Udager & Mehra, 2016).

Because of the loss of SDHB protein observed in tumor versus normal tissue, we considered the possibility of somatic alterations to the paternal *SDHA* wild‐type allele. Sequence analysis of an amplicon encompassing the mutated amino acid (Ala45/Ala3) in the germline and tumor DNA from the proband revealed no loss of heterozygosity (LOH) by deletion of the wild‐type allele in the tumor (see Supporting Information Data S4). Mutation analysis of the entire coding sequence could not be completed due to insufficient DNA, because of challenges associated with the presence of four non‐processed (i.e., with intact exon–intron junctions) *SDHA* pseudogenes in the genome and the technical limitations of amplification from FFPE material (see discussion in dos Santos, et al., 1991; Korpershoek, et al., 2011).

**Figure 4:**
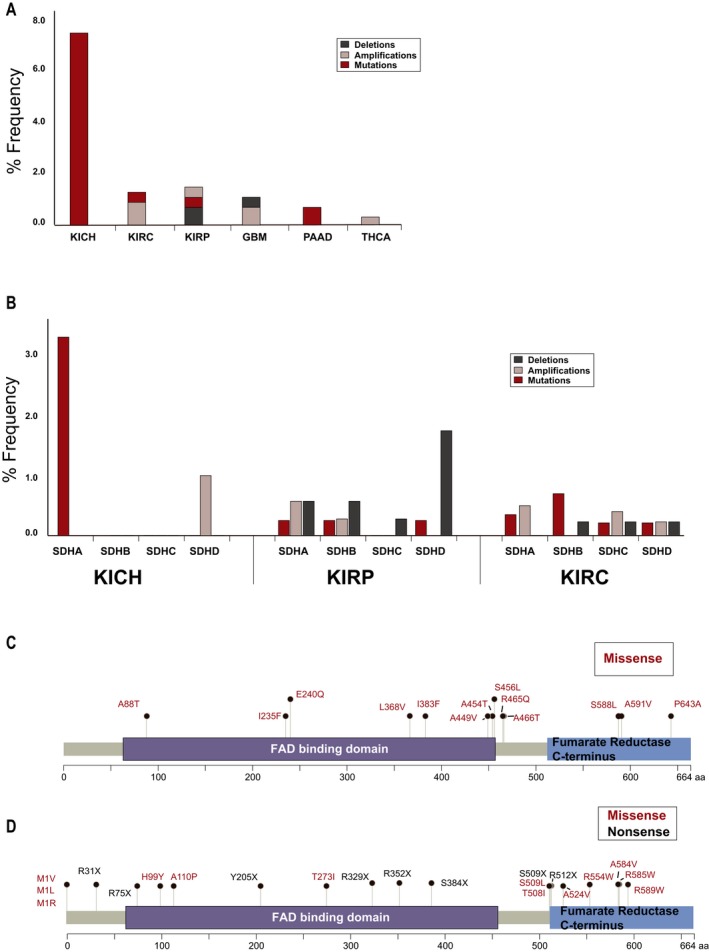
Genetic alterations reported for *SDH* complex genes in TCGA and other studies. (a) Somatic alterations in *SDHA* from most recent TCGA studies of cancers relevant to proband family history (downloaded from cbioportal.org). (b) Somatic alteration frequencies for *SDHA*, *SDHB*, *SDHC,* and *SDHD* across all renal cancer studies in cbioportal.org: KICH, KIRC, and KIRP. (c) Mapping of missense predicted‐to‐be damaging somatic *SDHA *mutations that were observed in most recent studies in cbioportal.org (also see Supporting Information Data S5). (d) Mapping of pathogenic and likely pathogenic germline missense and nonsense *SDHA* variants that have been reported in ClinVar (https://www.ncbi.nlm.nih.gov/clinvar/). GBM: glioblastoma multiforme; KICH: kidney chromophobe; KIRC: kidney renal clear cell carcinoma; KIRP: kidney renal papillary cell carcinoma; PAAD: pancreatic adenocarcinoma; THCA: thyroid carcinoma

We further explored in TCGA the profile of *SDHA *alterations (Figure 4a) in the various forms of RCC (clear cell [KIRC], papillary [KIRP], and chromophobe [KICH]), and in other cancers observed in the proband family (including pancreatic adenocarcinoma [PAAD], glioblastoma [GBM], and thyroid cancer [THCA]; note, basal cell carcinoma [BCC] is not represented in TCGA). In KICH, mutations in *SDHA* were observed in five of 66 cases. Overall, alterations in KIRC and KIRP were less commonly observed (six from over 450 cases, and four from over 280 cases, respectively). Low frequencies of *SDHA* alterations were also observed GBM, PAAD, and THCA (Figure 4a). We next explored the frequency of somatic alterations in *SDHx* genes in three renal cancer subtypes: KICH, KIRP, and KIRC, across multiple studies in cbioportal.org, which increased the total number of cases sequenced (Figure 4b, also see Supporting Information Data S5). Although mutations in *SDHA* were observed in KICH, no mutations were reported in the other three *SDHx* genes, although some *SDHD *amplifications were detected. In contrast, mutations and/or copy number variations were present at low levels in both KIRC and KIRP, for all *SDHx* genes. A schematic distribution of *SDHA* missense variants in cbioportal.org (Figure 4c) indicates most are located close to or within either the FAD‐binding domain or the C‐terminal fumarate reductase domain. Analysis of the missense mutations in TCGA using Annovar predicted 15 of the 16 somatic mutations was damaging through the consensus of multiple prediction programs (also see Supporting Information Data S5; cancer types relevant to the proband’s personal and family history were included in the analysis). Figure 4d schematically represents germline variants in *SDHA*. As germline mutations in *SDHA* are rare in RCC, we mapped all pathogenic or likely pathogenic missense and nonsense mutations reported in ClinVar in cancers and other diseases. Of these, none have been implicated in renal cancers. The sum of this analysis suggests that the Ala45Thr variant is plausibly pathogenic, however, further evaluation is needed to support pathogenicity.

### 3.3 Variants shared only by the siblings affected with cancer

Variants in *TGFB2* and *PARP1* were found in the proband and her affected brother, but not in the two unaffected siblings. The two affected individuals had compound heterozygosity, with two alterations only 8 nucleotides apart, in the 5′UTR of *TGFB2*, that may result in altered gene expression (Mignone, Gissi, Liuni, & Pesole, 2002; Supporting Information Data S6). *TGFB2* encodes TGFB2, which is a member of the TGFβ ligand superfamily, and is a potent regulator of cell differentiation and migration. TGFB2 action has been linked to formation of nephrons in development (Davies & Fisher, 2002; Plisov, et al., 2001). Polymorphisms in *TGFB2* have been associated with end‐stage renal disease (Ki, et al., 2015). TGFB2 signaling is relevant to pathogenesis of many cancers, including gliomas and pancreatic cancers (Dietrich, Dutoit, Tran Thang, & Walker, 2010; Hau, Jachimczak, Schlaier, & Bogdahn, 2011).

The *PARP1* variant Thr124Ala was inherited from the father (diagnosed with basal cell cancer at 78, and whose mother had brain cancer at 74). Poly(ADP‐ribose)polymerase‐1 (encoded by *PARP1*) is a chromatin‐associated enzyme that plays a role in the maintenance of genomic integrity, chromatin remodeling, and transcription control (Rajawat, Shukla, & Mishra, 2017). InterVar classified it as PM1 (pathogenic moderate) for location in a mutational hot spot or well‐studied functional domain without benign variation. T124 is located in the C_125_C_128_H_159_C_162_ zinc finger ZnF2 which strongly interacts with nicked or gapped DNA during the activation by genotoxic stress that results in cleavage of the ADP‐ribose moiety from NAD^+^ to generate poly(ADP‐ribosyl)ation of specific nuclear acceptor proteins, including histones, DNA polymerases, and PARP1 itself (Bossak, et al., 2015; Eustermann, et al., 2011).

**Figure 5:**
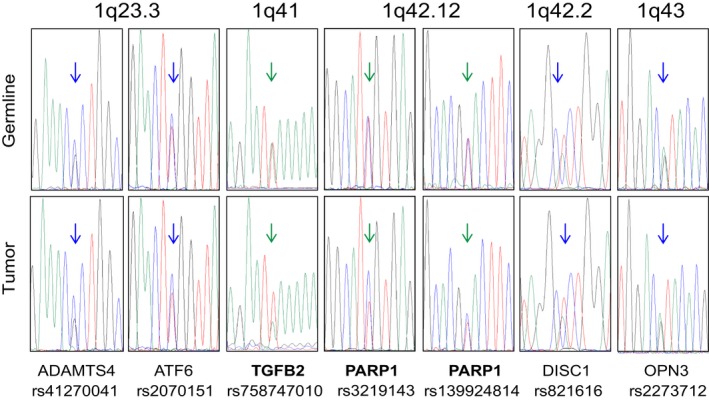
Sanger sequencing assessment detects allelic imbalance (AI) in *TGFB2, PARP1*, and other loci on chromosome 1q. PCR and Sanger sequencing for the indicated variant positions were carried out from germline (top) or tumor (bottom) DNA. For the variant in *TGFB2*, the A (green) allele is of maternal origin. For the variants in PARP1, the C (blue) alleles are of paternal origin. Green arrows indicate AI in the tumor, while the blue arrows point to lack of AI

Interestingly, comparative Sanger analysis of the DNA from blood and tumor DNA at the positions of rs7587470101 in *TGFB2*, and rs139924814 and rs3219143 in *PARP1,* revealed an over‐representation of the paternally inherited allele in the tumor (Figure 5). As these genes are located at 1q41 and 1q42.12, respectively, we tested the hypothesis that this allelic imbalance would represent a larger region of imbalance that might be relevant to renal cancer or SDH complex function. Candidate genes on chromosome 1q include *SDHC* located at 1q23.3, or *TOMM20* (coding for a mitochondrial translocase involved in SDHA import to the matrix) or *FH,* both located at 1q42.2. As the exome data did not provide heterozygous positions in these genes, markers in neighboring genes were used (ADAMTS4 and ATF6 for SDHC, DISC3 and OPN3 for TOMM20 and FH). No allelic imbalance was found at these positions (Figure 5).

### 3.4 Additional paternally inherited variants in the proband

The proband and her unaffected sister each carried an epidermal growth factor (*EGF*) variant, Ser16Thr, classified as PP2 (supporting pathogenic) for missense in a gene that has a low rate in benign missense variation, and in which missense variants are a common mechanism of disease in InterVar (Supporting Information Data S1). In the GnomAD database, this variant is overrepresented in the Ashkenazi Jewish population with an allele count of 15/10,146 versus 69/276,870 in the total population. Based on Signal 4.0, NCBI describes the preprotein (reference sequence NP_001954.2) as comprising a signal peptide (SP) at amino acids 1–14. In contrast, UniProt for reference sequence P0113 indicates a cleavage of a signal sequence after amino acid 22. For both predictions, an amino acid change at residue 16 plausibly affects insertion of the pro‐EGF precursor into the ER, and hence levels of secretion, which could cause multiple biological consequences, especially in the kidney that expresses high levels of EGF mRNA (Fisher, Salido, & Barajas, 1989). EGF is also important for thyroid metabolism (Mincione, et al., 2011), which may be significant, given the early thyroid cancer identified in the proband (Figure 1).

### 3.5 Additional maternally inherited variants

**Figure 6:**
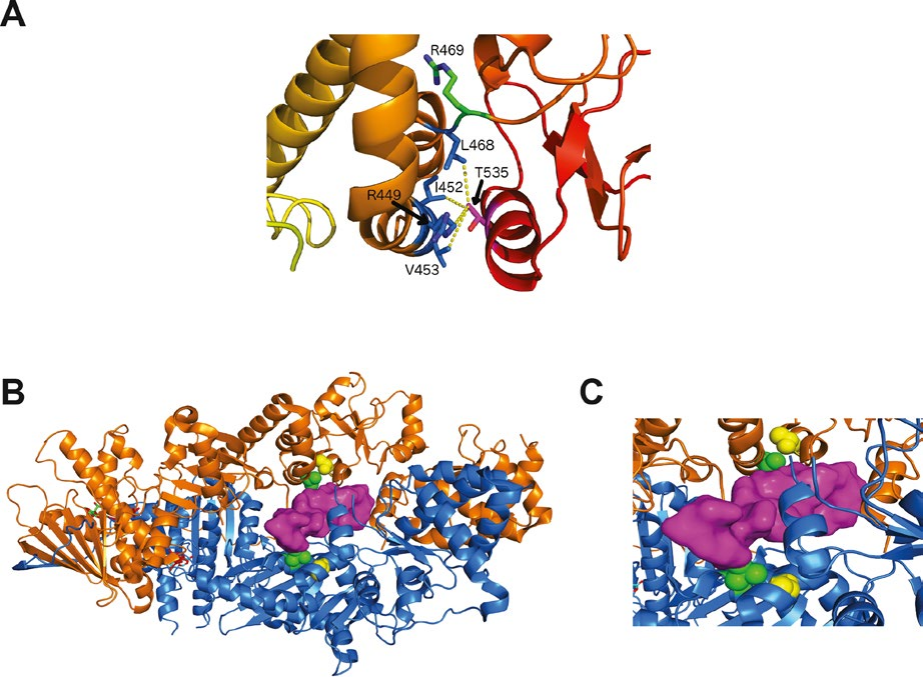
Structural analysis of TRAP1 Thr535Ser. (a) Hydrophobic contacts of the side chain of Thr535, located in helix 19, according to secondary structure numbering from Lavery et al. (Lavery, et al., 2014), with Arg449, Ile452, and Val453 of helix 14 and Leu468 of helix 15 of the middle domain (yellow lines). (b) Model of a peptide (in magenta) bound to the TRAP1 homodimer (in orange and blue). (c) Contact between Thr535 (yellow spheres) and Val453 (in green spheres) which interacts directly with a client peptide (in magenta). The model was built by superposing the middle domains of the TRAP1 homodimer onto those of HSP90 with CDK4 kinase domain bound. The folded kinase domains are not shown

The two affected siblings and the unaffected brother inherited a maternal *TRAP1* variant, Thr535Ser, with a PM1 classification. Consistent with the African descent of the mother noted in the *SDHA *variant analysis (see Supporting Information Appendix S1), this variant was overrepresented in African populations (Table 1). TRAP1 is a mitochondrial chaperone and key regulator of mitochondrial bioenergetics in tumor cells (Masgras, Sanchez‐Martin, Colombo, & Rasola, 2017). The Thr535Ser variant was recently described in a patient with Parkinson’s disease and characterized as damaging by functionality prediction and destabilizing by structural assessment (Fitzgerald, et al., 2017). Importantly, an examination of the X‐ray crystallographic structure of human TRAP1 indicates that the Thr535Ser substitution would disrupt hydrophobic interactions with the side chains of Arg449, Ile452, Val453, and Leu468 that stabilize the middle domain of the protein (Figure 6a). Further, Leu468 is particularly notable as a variant at an adjacent residue, Arg469His, has been identified as European founder mutation in CAKUT (Congenital Abnormalities of the Kidney and Urinary Tract) which accounts for approximately half of children with chronic kidney disease and is the most frequent cause of end‐stage renal disease in children in the US (Saisawat, et al., 2014). In analogy with HSP90, the TRAP1 homodimer has a channel through which clients may be bound. We used a structure of HSP90 bound to partially unfolded CDK4 to model how an unfolded client protein may bind to TRAP1 (Figure 6b,c). Although Thr535 (in yellow spheres) is not in direct contact with the client protein, it is in contact with Val453 (in green spheres) which interacts directly with the threaded peptide from CDK4 (in magenta surface). Thr535 is closer to the bound client in the model than Arg469, associated with CAKUT.

### 3.6 Maternally inherited variant found in the brother with kidney cancer at 35

In addition to the variants reported above, the affected brother inherited from his mother an *TRIB3* R36stop variant, also overrepresented in African population (Table 1). Tribbles pseudokinase 3, encoded by *TRIB3*, has a complex function that is tumor suppressive in some tissue types (Mondal, Mathur, & Chandra, 2016; Salazar, et al., 2015), but has also been linked to disease progression and therapeutic resistance for acute promyelocytic leukemia (Li, Wang, et al., 2017a). Specific pathways inhibited by TRIB3 include AKT/mTOR, influencing cell proliferation; while TRIB3 regulation of a set of transcription factors including ATF4, CHOP, C/EBPα, NF‐κB, and PPARγ can influence cell differentiation status and survival. The variant Q84R has been linked to metabolic disease and predisposition to diabetes and atherosclerosis (Prudente & Trischitta, 2015).

## 4 DISCUSSION

In this study of a female proband affected with thyroid and renal cancers, with a pedigree enriched for incidence of kidney and other cancers, we have identified several gene variants that are plausibly linked to kidney cancer risk. Among these, several categories of data support the potential significance of a rare, germline SDHA Ala45Thr variant in the DNA of the proband and her affected brother. Most directly, this evidence includes the presentation of a renal tumor negative for SDHB staining, with additional support from analysis of the literature and public databases. Although there are still a number of unanswered questions, these results generally support identification of this variant as a risk factor. However, further evidence is required to assign a confident classification of pathogenicity to the SDHA Ala45Thr variant. Pathogencity could be supported by evaluations that include assessment of functional impact, and variant segregation with disease (renal cancer or other) in the descendents of the studied proband or other families carrying the variant. For now, this variant is classified as a variant of uncertain significance. Several other variants of interest were also identified. In the proband and affected brother, the Thr124Ala variant in PARP1 and the Thr535Ser variant in TRAP1 are of particular interest because like SDHA, these proteins impact mitochondrial function, and defects in PARP1 and TRAP1 have been associated with cancer risk or CAKUT. Finally, other variants that may serve as primary or modifying factors for kidney cancer risk were identified in the proband, the brother, or both. Overall, our data are compatible with the idea that the elevated risk of cancer in the proband and her family may arise from the interaction of two or more rare variants.

Comprehensive reviews of *SDHA* variants over a large disease spectrum in various databases have been published (Bannon, et al., 2017; Casey, et al., 2017; Evenepoel, et al., 2015). To date, there have been few reports of *SDHA* mutations in sporadic renal cancer: for example, a 17 kbp homozygous deletion leading to the loss of 9 exons of SDHA (Yakirevich, et al., 2015), a heterozygous germline mutation in the initiation codon (Jiang, et al., 2015), a splice site deletion (Ozluk, et al., 2015), and a combined germline/somatic biallelic loss (McEvoy, et al., 2018). The SDHA Ala45Thr variant was previously reported in a case of thoracic paraganglioma (Casey, et al., 2017) but was largely uncharacterized. Another rare variant, at the adjacent position in the primary structure (Lys46Glu), has been reported in a case of abdominal paraganglioma (Casey, et al., 2017).

The presence of the *SDHA* variant in healthy individuals (mother, unaffected brother) suggested that this variant may be considered as incompletely penetrant, rather than strongly predisposing. Low penetrance of *SDHA* variants with carriers escaping the development of clinical symptoms has been suggested (Casey, et al., 2017; Korpershoek, et al., 2011). Whether the proband’s *SDHA* allele of paternal origin also carried a deleterious somatic mutation in the tumor could not be determined due to lack of tumor material, leaving open the possibility that loss of SDHB expression we observed in the patient’s tumor resulted from deficient *SDHA* functionality rather than the more classical two‐hit tumor suppressor mechanism. In a study of SDHx, in 85 tumors of various types with information on the nature of the second hit, inactivation of the wild‐type allele by loss of heterozygosity and somatic mutations occurred with frequency of 73% and 14%, respectively (Evenepoel, et al., 2015). In the case of SDH‐deficient renal carcinomas, a damaging variant in a SDHx gene seems to most commonly be the cause of lost expression of SDHB (Gill, et al., 2014), enforcing the conclusion that Ala45Thr is a plausible risk factor.

Germline variants or somatic mutations in genes of the SDH complex cause a cascade of molecular events that promote tumorigenesis, mainly through the accumulation of the oncometabolite succinate, and influence oxidative phosphorylation (Zhao, Mu, & You, 2017). These activities raise the possibility that the specific combination of identified variants may have elevated risk in the affected family members, based on dual or triple insult to these processes. In this context, the *TRAP1* and *PARP1* variants are of particular interest. TRAP1 is a key regulator of mitochondrial bioenergetics in tumor cells. TRAP1 can decrease SDH enzymatic activity, thus resulting in high concentration of succinate, and contributing to tumor cell survival in the stress conditions of neoplastic growth (Masgras, et al., 2017). Our structural characterization of the TRAP1 variant Thr535Ser suggests that it potentially affects the chaperone activity of TRAP1, involved in quality control of matrix proteins. Interestingly, the undetectable activity of SDHB in the renal tumor‐derived cell line UOK269, carrying the SDHB Arg46Gln variant, has been linked to the disrupted binding to another co‐chaperone, HSC20 (Saxena, et al., 2016).

Of further interest, the paternally inherited *PARP1* Thr124Ala was shared by the two affected siblings. Poly(ADP‐ribose)polymerase 1 (encoded by *PARP1*) is best known for a role in the maintenance of genomic integrity, chromatin remodeling, and transcription control (Rajawat, et al., 2017), but also has a role in mitochondrial homeostasis (Vida, Marton, Miko, & Bai, 2017). In its repair function, PARP1 becomes activated after binding to single‐ and double‐stranded breaks through the zinc fingers ZnF1 and ZnF2, where Thr124 is located. The crystal structure of the human PARP1‐DNA‐binding domain bound to a DNA duplex also showed the adjacent residues 120–123 interact with the minor groove (Ali, et al., 2012). Accumulated succinate from SDH deficiency drives an intracellular ROS generation leading to excessive DNA oxidation. Response to ROS‐associated DNA damage may be greater in the affected siblings who inherited the paternal Thr124Ala PARP1 variant than in the unaffected mother. The tumor of the proband presented an allelic imbalance with over‐representation of the Thr124Ala allele. 1q41‐q42, the location of the PARP1 and TGFB2 genes, is known to be involved in a microdeletion syndrome causing developmental abnormalities (Shaffer, et al., 2007). Interestingly, mutations and allelic imbalance were found to be two mechanisms targeting PARP1 in diffuse large B cell lymphomas (de Miranda, et al., 2013), which may be relevant for the proband and her affected brother.

TRIB3 has been proposed to act as guardian of the genome by protection of nuclear DNA from cytidine deamination by APOBEC3A (Aynaud, et al., 2012), Speculatively, considering this proposed role, response to succinate accumulation may be even further altered in the brother carrying the early truncating variant *TRIB3* R36stop, in addition to *SDHA* and *PARP1* variants, possibly explaining the onset at age 35. The variants in *EGF* and *TGFB2* are more challenging to interpret at this point. Their report here may shed light on further studies by us or others with related findings. Finally, recognizing the patient has a pattern of maternally inherited alleles suggesting some African heritage may also be of importance, given extensive differences in African versus European inherited mitochondrial DNA haplogroups (Kenney, et al., 2014), and the recognized relevance of mitochondrial haplotype for renal cancer (Booker, et al., 2006).

Considering the number of mitochondrial disorders and hereditary tumors associated with *SDHx* genes, knowledge of *SDHx* carrier status is important for the clinician (Hall, Forman, & Obeid, 2017). While the basis of classification of RCCs is moving from morphological to molecular criteria with next‐generation sequencing in clinical oncology for better patients triage toward successful therapies, we recognize the current paucity of complete sets of data (morphology, SDHB IHC, SDHx genomic screening, and robust profiling of rationally selected candidate contributing variants). Renal tumors with a homozygous deletion of 9 exons of *SDHA* or a splice deletion also been reported in some but not all studies to have histological signs of SDH‐deficiency including characteristic cytoplasmic vacuoles and inclusions (contrast (Ozluk, et al., 2015; Yakirevich, et al., 2015) with (Li, Reuter, et al., 2017b)). The proband from the tumor in this study lacks these cytoplasmic vacuoles and inclusions, and together with work of Li and colleagues, our analysis suggests these distinctive morphological features should not be solely used for triage for genomic SDHx testing. This work emphasizes the need for multidisciplinary approach for proper variant data interpretation and support of the clinician.

## ETHICAL APPROVAL

The analysis performed and publication of deidentified information was under the approval of the Fox Chase Cancer Center (FCCC) Institutional Review Board Committee protocol number 14–831, and the family provided written informed consent to the FCCC Risk Assessment Program Registry.

## CONFLICT OF INTEREST

None declared.

## Data Availability

For the data generated, consent was not obtained for data sharing in publicly accessible databases. ClinVar submission can be viewed at the following address: https://www.ncbi.nlm.nih.gov/clinvar/submitters/506523/.
