# Supplementary figures and images for: Interaction of germline variants in a family with a history of early‐onset clear cell renal cell carcinoma
Source: Mol Genet Genomic Med. 2019 Jan 24;7(3):e556. doi: 10.1002/mgg3.556 (PMC6418363; doi:10.1002/mgg3.556)

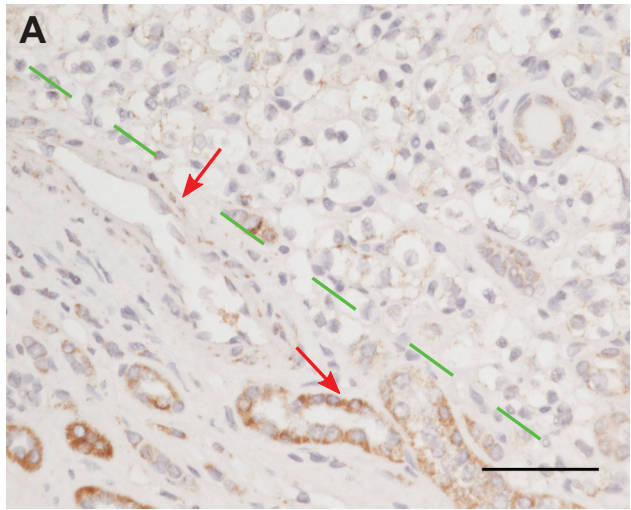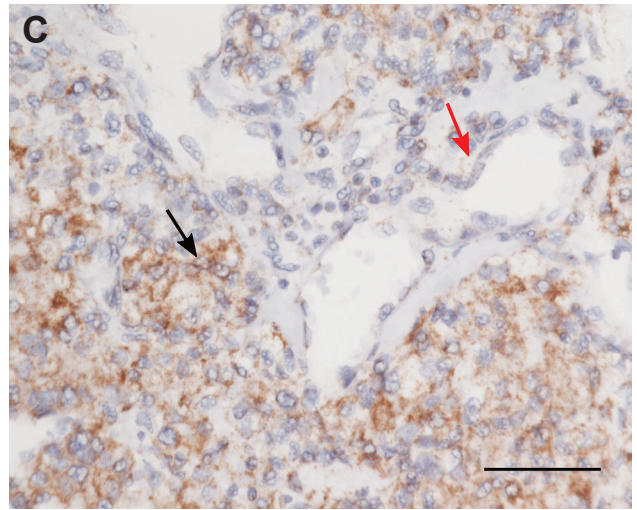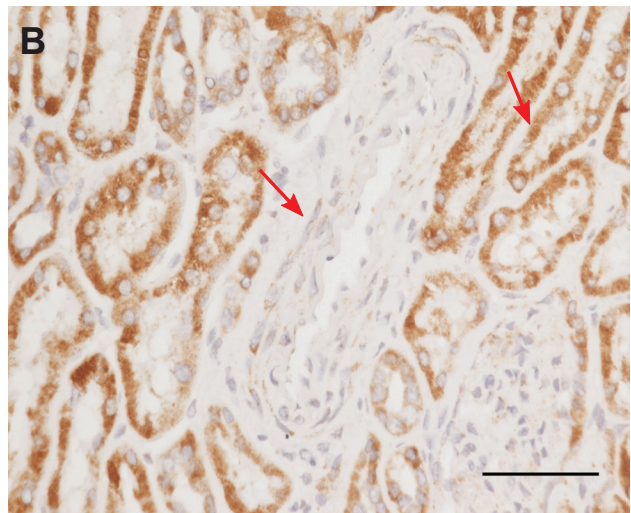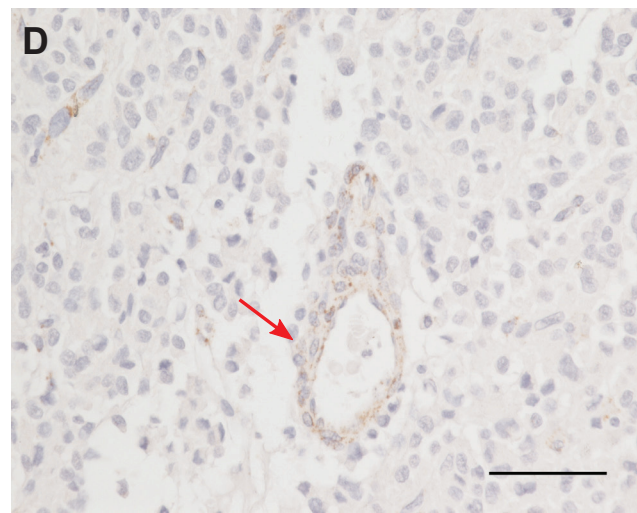

Supplement: Supplementary file 1 [file MGG3-7-na-s001.pdf]

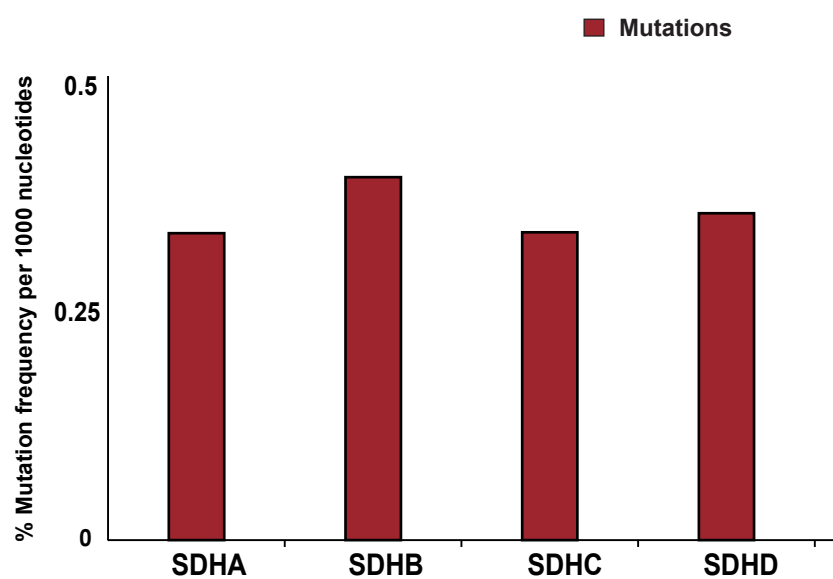

Supplement: Supplementary file 2 [file MGG3-7-na-s002.pdf]
