# Supplementary material for: Interaction of germline variants in a family with a history of early‐onset clear cell renal cell carcinoma
Source: Mol Genet Genomic Med. 2019 Jan 24;7(3):e556. doi: 10.1002/mgg3.556 (PMC6418363; doi:10.1002/mgg3.556)

**Supplementary File 2: Sanger validation for the selected variants identified by exome sequencing.**

**PARP1** [**rs139924814**](https://www.ncbi.nlm.nih.gov/projects/SNP/snp_ref.cgi?rs=139924814)ATCTTCTCCATACACCCCTTGCACG[C/T]ACTTCTGTTGGACTTGGCATACTCT

Forward: CGTTGAGCTAGCACCCTAAA, Reverse CTGTTTGATTCTCCAGGCAAAG, 242 bp

Proband:


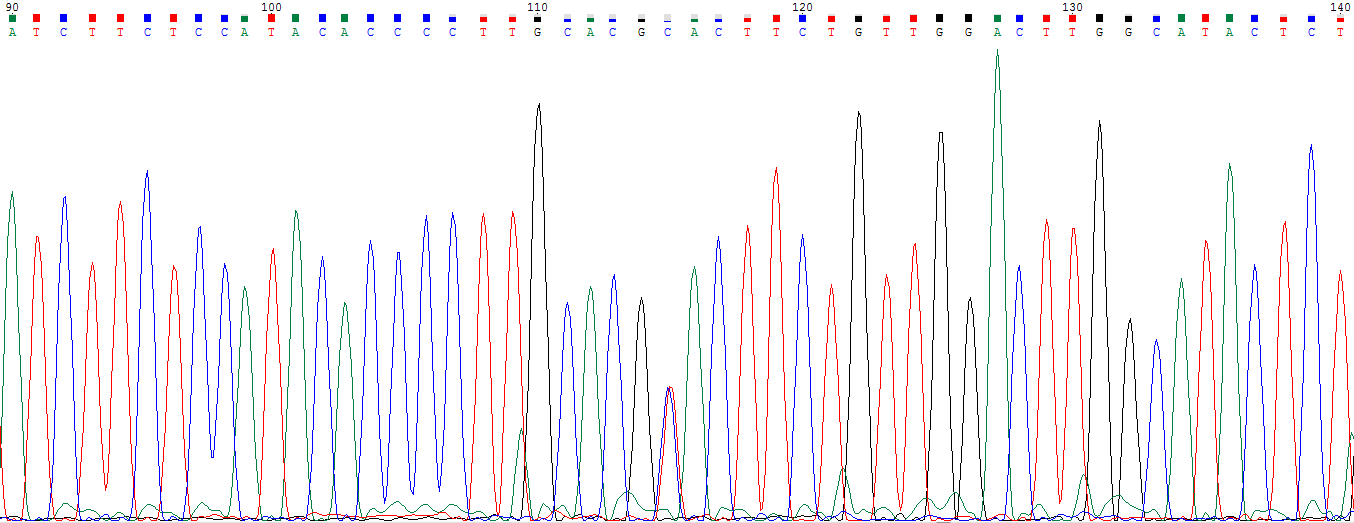


**EGF** [**rs200394315**](https://www.ncbi.nlm.nih.gov/projects/SNP/snp_ref.cgi?rs=200394315) CTGTTGCCAGTAGTTTCAAAATTTA[C/G]TTTTGTTAGTCTCTCAGCACCGCAG

Forward: AAGAGCTTGGAGGACAACAG, Reverse: GAGTACTTACCCACACAAGTAGAA, 300 bp

Proband


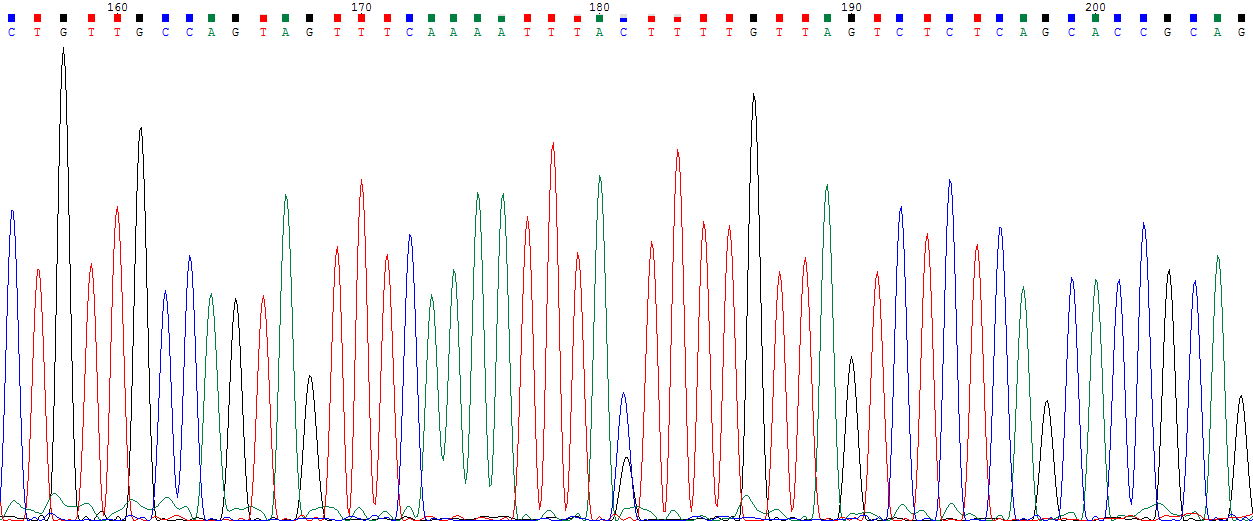


**TRAP1** [**rs77440336**](https://www.ncbi.nlm.nih.gov/projects/SNP/snp_ref.cgi?rs=77440336)

GTCAAACTCACGAAGGTGCAGCAGG[C/G]TGAGCTCATCAAACTGCTCAAAGCA

Forward: CCTCAAACTTCTCCTCCTTGTAG, Reverse: AGACCCAGGTGTCGTAGTAA, 295 bp

Proband


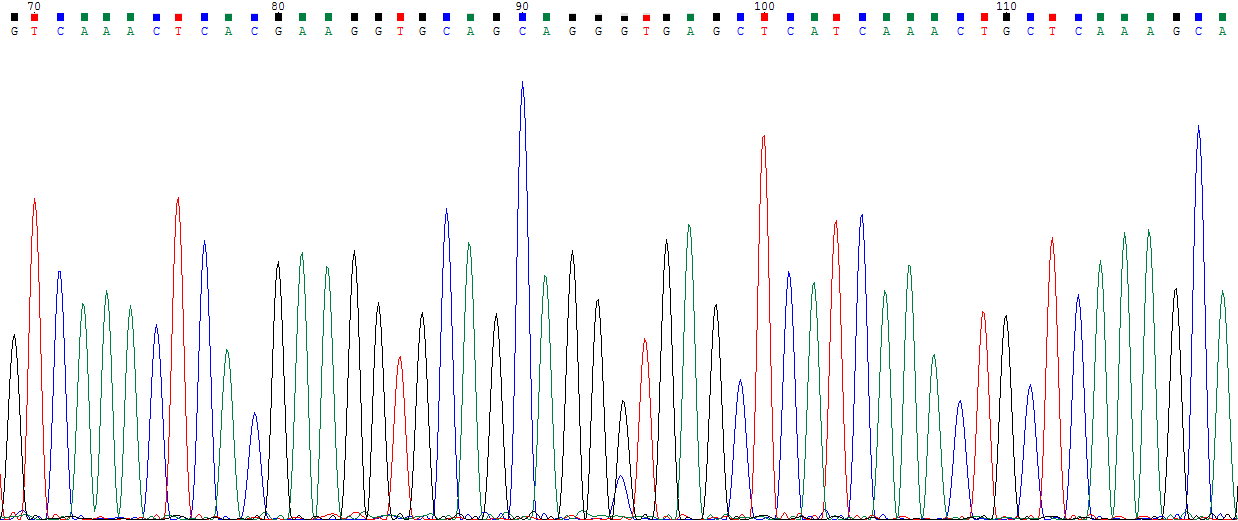


**TRIB3** [**rs150841542**](https://www.ncbi.nlm.nih.gov/projects/SNP/snp_ref.cgi?rs=150841542)

CGAGCGTCCCGTCCAGAAACGAGCT[C/T]GAAGTGGGCCCCAGCCCAGACTGCC

Forward: AGCAGTCTCACTTTAGTGCTTT, Reverse: AGGACATAGGGCCCAAGA, 257 bp

Affected brother


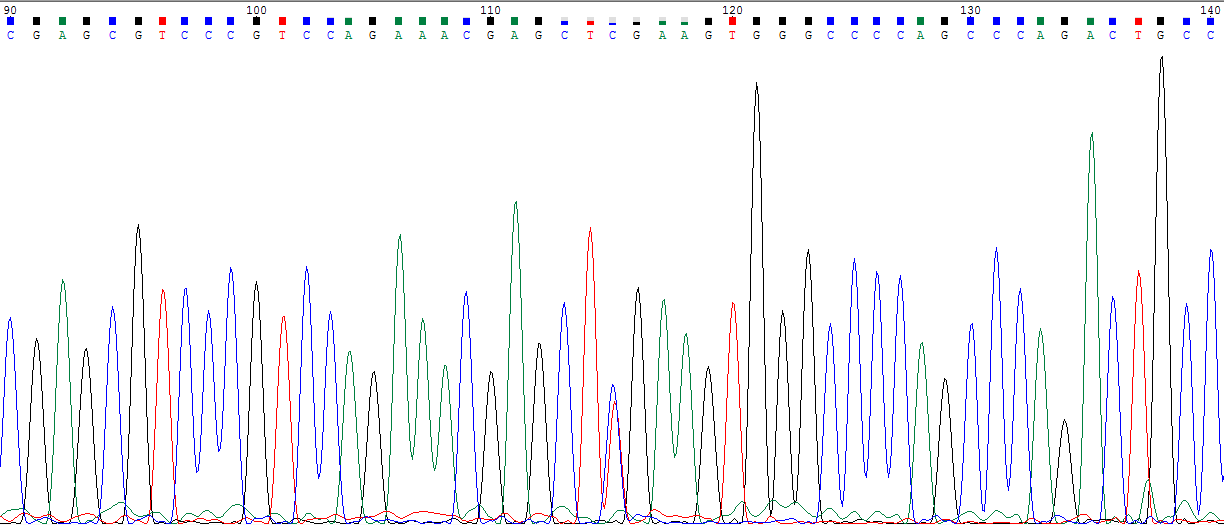


**TGFB2** [**rs200186989**](https://www.ncbi.nlm.nih.gov/projects/SNP/snp_ref.cgi?rs=200186989)

CTATACTTTGAGAATTGTTGATTTC[-/T]TTTTTTTATTCTGACTTTTAAAAAC

Forward: TTGGGCATTGACTAGATTGTTTG, Reverse: ATGAACTGGTCCATATCGAGTG, 257 bp

Father (sequenced with reverse primer)


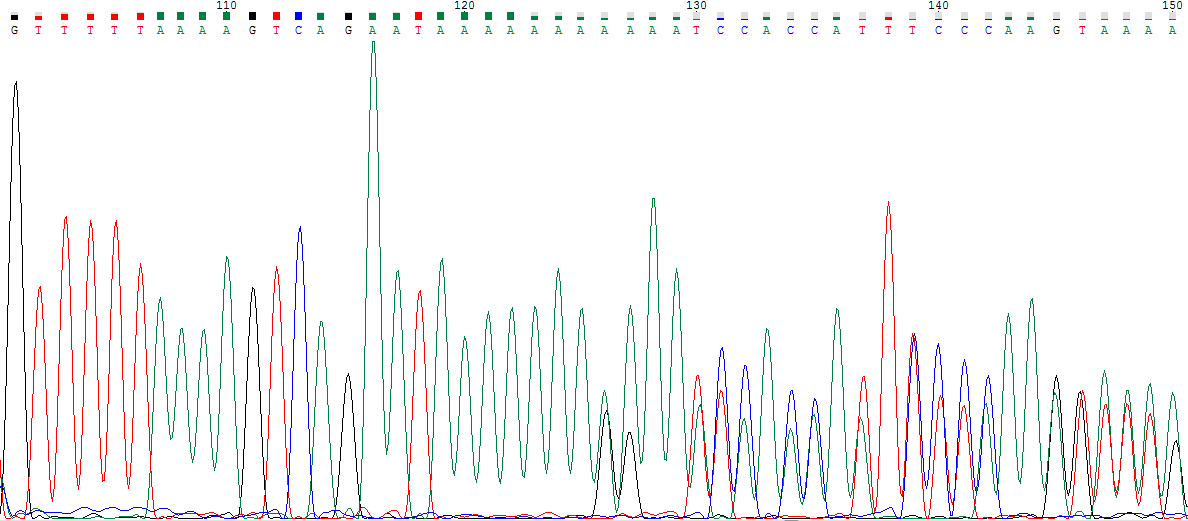


**TGFB2** [**rs758747010**](https://www.ncbi.nlm.nih.gov/projects/SNP/snp_ref.cgi?rs=758747010)

TGAGAATTGTTGATTTCTTTTTTTT[-/A]ATTCTGACTTTTAAAAACAACTTTT

Same primers as above

Mother (sequenced with reverse primer)


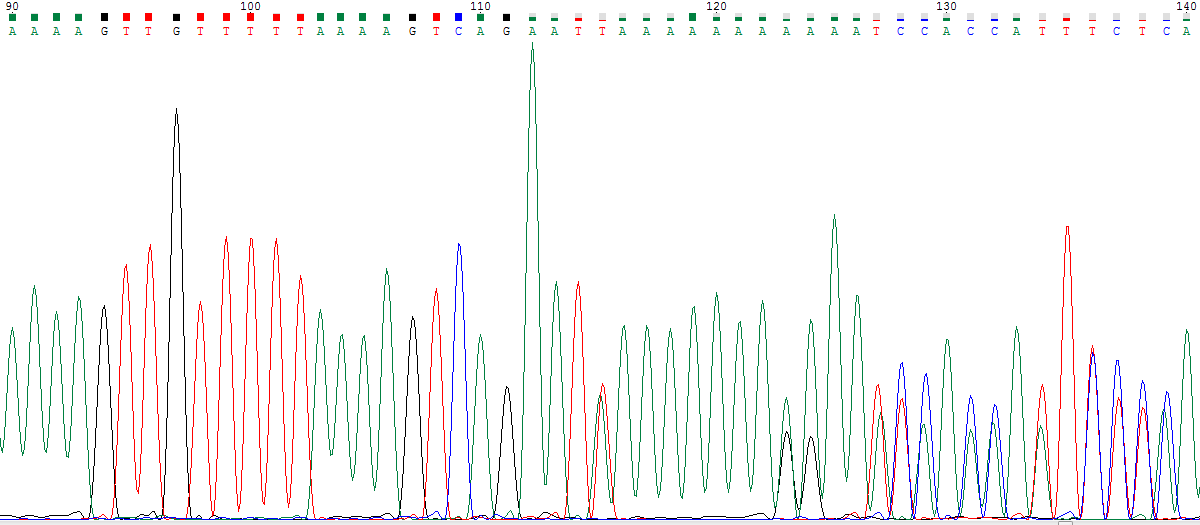


[**TGFB2** **rs200186989**](https://www.ncbi.nlm.nih.gov/projects/SNP/snp_ref.cgi?rs=200186989)

CTATACTTTGAGAATTGTTGATTTC[-/T]TTTTTTTATTCTGACTTTTAAAAAC

**and** [**rs758747010**](https://www.ncbi.nlm.nih.gov/projects/SNP/snp_ref.cgi?rs=758747010)

TGAGAATTGTTGATTTCTTTTTTTT[-/A]ATTCTGACTTTTAAAAACAACTTTT

Same primers as above

Proband (sequenced with reverse primer)


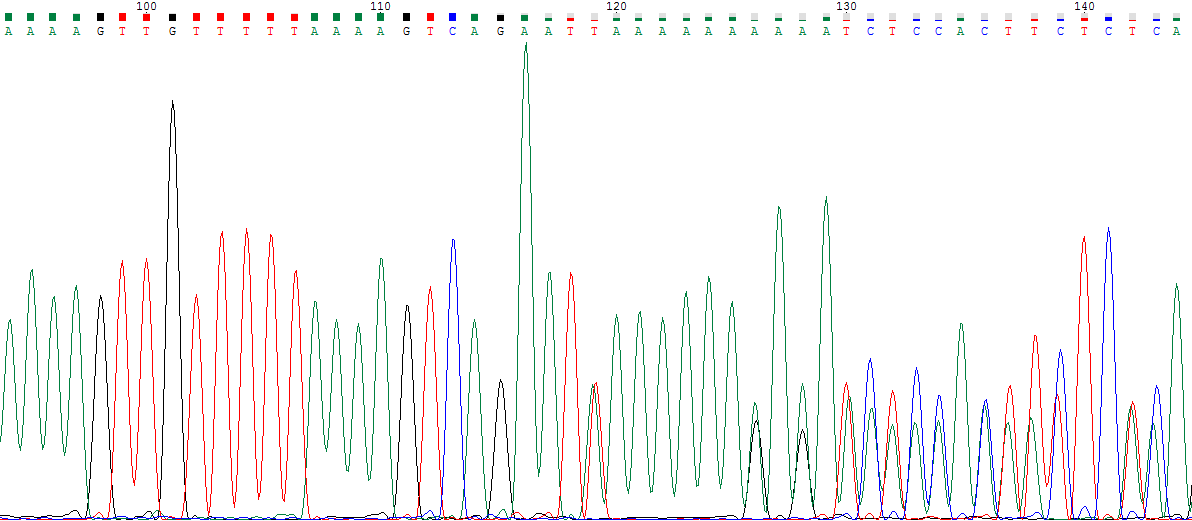

Supplement: Supplementary file 4 [file MGG3-7-na-s004.doc]
