# Supplementary material for: Interaction of germline variants in a family with a history of early‐onset clear cell renal cell carcinoma
Source: Mol Genet Genomic Med. 2019 Jan 24;7(3):e556. doi: 10.1002/mgg3.556 (PMC6418363; doi:10.1002/mgg3.556)

**Supplementary File 4: Sanger sequencing analysis of SDHA rs140736646 (Ala45Thr) from proband blood and tumor.**

SDHA, rs140736646

TGTTGATGGGAACAAGAGGGCATCT[A/G]CTAAAGTTTCAGATTCCGTAAGTTC

Primers: CAGTTTGCAAGGGGAAATTACT, AGCATGAACTTACGGAATCTGA

Amplification from blood
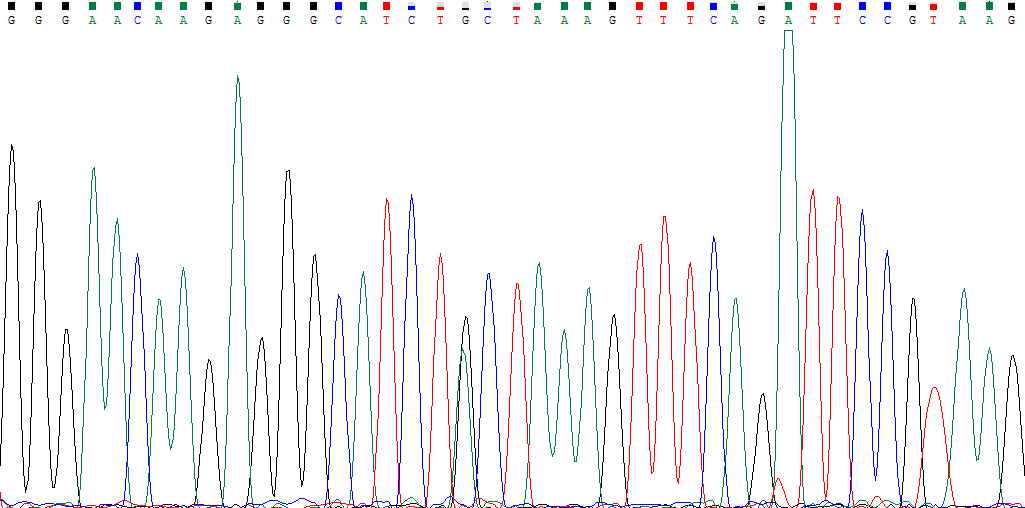


Amplification from tumor


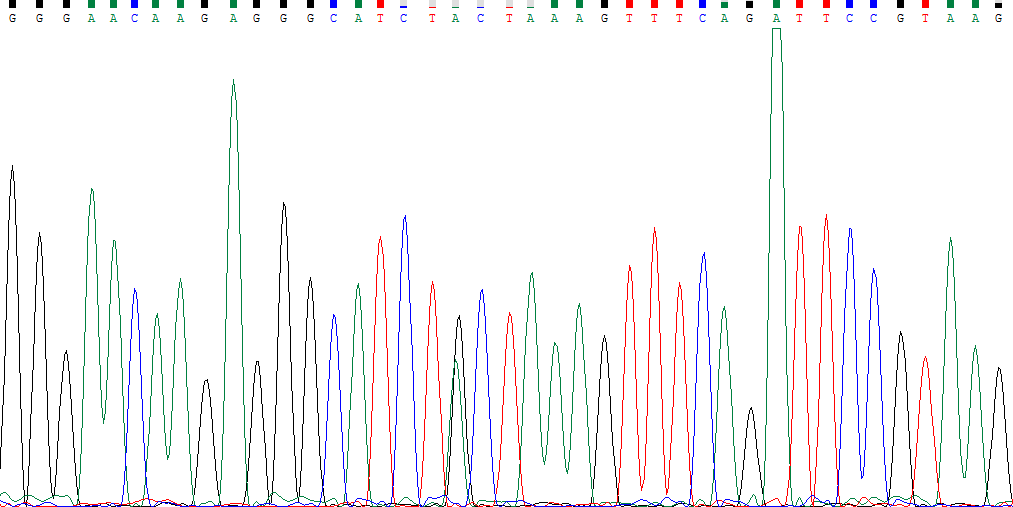

Supplement: Supplementary file 6 [file MGG3-7-na-s006.docx]
