# Supplementary material for: Interaction of germline variants in a family with a history of early‐onset clear cell renal cell carcinoma
Source: Mol Genet Genomic Med. 2019 Jan 24;7(3):e556. doi: 10.1002/mgg3.556 (PMC6418363; doi:10.1002/mgg3.556)
Supplement: Supplementary file 8 [file MGG3-7-na-s008.docx]

**Supplementary File 5: 5’UTR TGFB2 variants**

Partial NCBI Reference Sequence (NM_003238.4) showing location of the start codon (ATG) and alterations in father and mother DNAs

Father: T deletion

[rs200186989](https://www.ncbi.nlm.nih.gov/projects/SNP/snp_ref.cgi?rs=200186989): CTATACTTTGAGAATTGTTGATTTC[-/T]TTTTTTTATTCTGACTTT

Mother: A insertion

[rs758747010](https://www.ncbi.nlm.nih.gov/projects/SNP/snp_ref.cgi?rs=758747010): TGAGAATTGTTGATTTCTTTTTTTT[-/A]ATTCTGACTTTTAAAAA

TCAAAAACAACAACAACAAAAAACCAAACAACTCTCCTTGATCTATACTTTGAGAATTGTTGATTTC↓TTTTTTTT↓ATTCTGACTTTTAAAAACAACTTTTTTTTCCACTTTTTTAAAAA**ATG**CACTACTGTGTGCTGAGCGCTTTTCTGATCCTGCATCTGGTCACGGTCGCGCTCAGCCTGTCTACCTGCAGCACACTCG

Analysis with RNA Analyzer <http://rnaanalyzer.bioapps.biozentrum.uni-wuerzburg.de/> showing an additional SMSITES or snRNP-binding motif created by the A insertion in the maternal DNA

UCAAAAACAACAACAACAAAAAACCAAACAACUCUCCUUGAUCUAUACUUUGAGAAUUGUUGAUUUC(U)UUUUUUUAUUCUGACUUUUAAAAACAACUUUUUUUUCCACUUUUUUAAAAA**AUG**CACUACUGUGUGCUGAGCGCUUUUCUGAUCCUGCAUCUGGUCACGGUCGCGCUCAGCCUGUCUACCUGCAGCACACUCG

CAACTTTT

UCAAAAACAACAACAACAAAAAACCAAACAACUCUCCUUGAUCUAUACUUUGAGAAUUGUUGAUUUCUUUUUUUUAAUUCUGACUUUUAAAAACAACUUUUUUUUCCACUUUUUUAAAAA**AUG**CACUACUGUGUGCUGAGCGCUUUUCUGAUCCUGCAUCUGGUCACGGUCGCGCUCAGCCUGUCUACCUGCAGCACACUCG
