# Supplementary material for: Interaction of germline variants in a family with a history of early‐onset clear cell renal cell carcinoma
Source: Mol Genet Genomic Med. 2019 Jan 24;7(3):e556. doi: 10.1002/mgg3.556 (PMC6418363; doi:10.1002/mgg3.556)
Supplement: Supplementary file 10 [file MGG3-7-na-s010.docx]

**Supplementary Data and Discussion.**

**SDHA variant.** According to the gnomAD database**,** SDHAAla45Thr is present in 1 in 651 individuals of European ancestry. While it could be argued that variants with low penetrance have questionable diagnostic utility, we note that assessments of the impact of such variants for risk have high discordance rates in ClinVar (Yang et al. 2017), especially if they are poorly annotated, and represents a challenge for the clinician.

In this study, while the proband disclosed a German ancestry, the analysis of the full exome data report for *SDHA* indicated that the mother also carried, in homozygous state, variants linked to African ancestry. As discussed below, these variants are not rare, and have been previously characterized as having no effect when studied in a yeast model (Bannon et al. 2017), and so were viewed as not likely to be pathogenic. However, considering the need to address the challenge of higher incidence of cancer in populations of African ancestry, possibly through better knowledge of specific characteristics of their DNA variant profile (Ragin et al. 2017), we further evaluated data supporting the idea that maternal *SDHA* allele of African origin.

**African origin of the maternal SDHA allele.**  Suppl. File 3 lists the coordinates of 65 locations differing from the reference genome from the exome data from the proband’s mother with distribution in total and African populations in ExAC and GnomAD databases. Out of the 20 variants for which data are available, 14 showed higher frequencies (~0.50 over ~0.15) in the African population than in the total population. With such high allelic frequencies, the variants are not expected to cause disease, but they may not be just dismissed as they may influence the course of disease or treatment, as exemplified by the African-specific variant Pro47Ser in TP53, which has reported to affect chemosensitivity (Basu et al. 2016). Our data identified the mother as carrier of the missense variants Tyr629Phe (rs6960) and Val657Ile (rs6962) and the silent variants at codons Ala103, Asn228, Ser346, Thr560 and Val644 which have been described as belonging to the same haplotype (Baysal et al. 2007) . Also described in this later study was the excess of *SDHA* variants in African Americans. Consistent with it, is the finding that the father’s DNA had only 12 entries for SDHA in the exome report while the mother had 65 (data not shown).

As reviewed by Rotimi et. al, exposure of Africans to fatal pathogens, such as *Plasmodium* *falciparum,* Lassa virus, and *Trypanosoma brucei rhodesiense*, has resulted in elevated frequencies of alleles that confer survival advantages for infectious diseases but are not always adapted for modern environments (Rotimi et al. 2017). Examples of loci protective against malaria that are associated with inherited diseases include HBB for sickle cell, or HBG1 for hypertension (Rotimi et al. 2017). Balancing selection refers to the maintenance of distinct alleles because they provide an advantage. It was described for *SDHA* in 2007 (Baysal et al. 2007). It was hypothesized that environment factors such as the plant and fungal toxin 3-nitropropionic acid 3-NPA or other pathogens prevalent in Africa may have contributed to the selection of *SDHA* variants that might have different roles in the regulation of oxygen homeostasis by the SDH complex. 3-NPA has been shown to act as an irreversible inactivator of avian SDHA by forming a covalent adduct with the side chain of Arg297 in the mature protein (Huang et al. 2006) . The equivalent location of this amino acid in the human SDHA preprotein is Arg340. There is no report of mutation of this residue. African-specific variants include: 1- Tyr629Phe: Tyr629 is highly conserved through evolution, and is located near the SIRT3 acetylation sites at residues 624 and 633 (Finley et al. 2011). SIRT3 loss results in decreased SDH enzyme activity, suggesting that SIRT3 may be an important physiological regulator of SDH activity (Finley et al. 2011) . 2-Val657Ile: Val657 is located in the carboxyterminal 13 amino acids of SDHA. Truncation to remove these 13 amino acids from the yeast sdh1 protein (orthologous to SDHA) has been shown to impair flavination (Robinson and Lemire 1996; Kim et al. 2012). 3-Glu640Gly: this variant was recently been described as non-segregating with thyroid carcinoma in a large Brazilian family (Accordi et al. 2016). Functional studies in a yeast model found no effect for the two missense variants Tyr629Phe and Val657Ile (Bannon et al. 2017).

**Presequence processing.** Consistent with the description of the mitochondrial protein import machinery as a crucial regulatory hub for metabolism (Harbauer et al. 2014), the location of the variant near the SDHA presequence suggested that Ala45Thr may alter the maturation of the protein to its functional form in the mitochondrial matrix. As described in (Vaca Jacome et al. 2015)) the characterization of the human “N-terminome” is still an emerging challenge. Only a small fraction of mitochondrial precursors have been experimentally analyzed for cleavage, limiting the information for substrate specificity (Burkhart et al. 2015). The identification of multiple unique proteases involved in cleavage underlines that the process is not as simple as originally anticipated (Poveda-Huertes et al. 2017). The possible critical location of a variant near a protein processing site may not be taken into consideration by algorithms predicting damaging impact. The lack of sequence conservation in the presequences and adjacent regions previously reported for various proteins (Calvo et al. 2017) and documented for SDHA in Figure 2 leads to poor scores by predictors such as SIFT that are mainly based on features such as amino acid conservation and biochemical properties of amino acids substitutions. Newer prediction methods also use basic genomic and protein annotations from ENCODE and Ensembl, that may include proximity to the presequence. Hence, not surprisingly, the Ala45Thr variant was predicted to be benign or tolerated by the older protein damage predictors SIFT, PolyPhen2, LRT, FATHMM, PROVEAN, MetaSVM, and M-CAP, but as damaging by Mutation Taster and Fathmm-MKL, and likely pathogenic by Mutation Taster (Suppl. File 1). We believe that at this point, there is not enough information on the identification of the N-termini and their processing for the pathogenicity predictors for amino-acids in or near the mitochondrial pre-sequence to be deemed highly reliable. The current example emphasizes the importance of not depending solely on such scoring systems for assessment of the damaging potential of variants.

After cleavage of the presequence (Fig 2C), Ala45 becomes Ala3 in the mature protein. While there is no experimental structure of human SDHA, there are several structures of its orthologs in the Protein Data Bank (PDB), including structures of the pig (Inaoka et al. 2015) and chicken (Huang et al. 2006) proteins. In each of these structures, the protein construct that was crystallized begins at the N-terminus of the mature protein (Ala43 of the preprotein). As the first 7-9 residues were not observed in the electron density map and not modeled in the coordinates deposited in the PDB, the functional consequences of the Ala45Thr (Ala3Thr of the mature protein) cannot be inferred from these structures.

As shown in Figure 2A, the SDHA RX(↓)(F/L/I)XX(T/S/G)XXXX(↓) motif, where arrows indicate successive cleavages by MMP and Oct1, starts at position -12 rather than the canonical -10. This implies that the Oct1 product has to be processed further by matrix proteases to generate the mature protein starting as ASAKV. At this point, there is no known candidate which makes it challenging to conclude whether the mutation Ala3Thr has consequences on cleavage is unknown. Reports where a variation located near the cleavage site has incidence on cleavage include the variant Ala673Thr of APP that confers protection to Alzheimer’s disease: the substitution which is located at position 2 in the amyloid peptide generated by proteolytic cleavage site and has been shown to inhibit BACE1 cleavage (Jonsson et al. 2012). For some mitochondrial matrix proteins, the process of presequence trimming has been elucidated. For example, in yeast, the aminopeptidase ICP55 removes three N-terminal residues from mitochondrial NFS1 (Naamati et al. 2009) through three consecutive steps of hydrolysis of Tyr-↓-Ser, Ser-↓-Pro and Pro-↓-Pro (Singh et al. 2017). More work is needed for SDHA. The Ala45Thr variant could also influence the recognition by the translocase responsible for mitochondrial import (Schleiff et al. 1999; Zhang et al. 2013), acting to regulate import either of the pre-protein, or (as Ala3Thr) of the processed mature protein. Finally, the Ala45Thr variant could also affect stability of SDHA, as the mature protein is subject to mitochondrial quality control (Voos et al. 2016; Poveda-Huertes et al. 2017) after import, with misfolded proteins degraded by proteolysis by LONM and other proteases. This quality control includes interaction of SDHA with the chaperone TRAP1 (Tumor necrosis factor receptor associated protein 1), discussed further in the main text as particularly relevant in this family (Sciacovelli et al. 2013).

**Supplementary References.**

Accordi ED, Xekouki P, Azevedo B, de Alexandre RB, Frasson C, Gantzel SM, Papadakis GZ, Angelousi A, Stratakis CA, Sotomaior VS et al. 2016. Familiar Papillary Thyroid Carcinoma in a Large Brazilian Family Is Not Associated with Succinate Dehydrogenase Defects. *Eur Thyroid J* **5**: 94-99.

Bannon AE, Kent J, Forquer I, Town A, Klug LR, McCann K, Beadling C, Harismendy O, Sicklick JK, Corless C et al. 2017. Biochemical, Molecular, and Clinical Characterization of Succinate Dehydrogenase Subunit A Variants of Unknown Significance. *Clin Cancer Res* **23**: 6733-6743.

Basu S, Barnoud T, Kung CP, Reiss M, Murphy ME. 2016. The African-specific S47 polymorphism of p53 alters chemosensitivity. *Cell Cycle* **15**: 2557-2560.

Baysal BE, Lawrence EC, Ferrell RE. 2007. Sequence variation in human succinate dehydrogenase genes: evidence for long-term balancing selection on SDHA. *BMC Biol* **5**: 12.

Burkhart JM, Taskin AA, Zahedi RP, Vogtle FN. 2015. Quantitative Profiling for Substrates of the Mitochondrial Presequence Processing Protease Reveals a Set of Nonsubstrate Proteins Increased upon Proteotoxic Stress. *J Proteome Res* **14**: 4550-4563.

Calvo SE, Julien O, Clauser KR, Shen H, Kamer KJ, Wells JA, Mootha VK. 2017. Comparative Analysis of Mitochondrial N-Termini from Mouse, Human, and Yeast. *Mol Cell Proteomics* **16**: 512-523.

Cerami E, Gao J, Dogrusoz U, Gross BE, Sumer SO, Aksoy BA, Jacobsen A, Byrne CJ, Heuer ML, Larsson E et al. 2012. The cBio cancer genomics portal: an open platform for exploring multidimensional cancer genomics data. *Cancer Discov* **2**: 401-404.

Finley LW, Haas W, Desquiret-Dumas V, Wallace DC, Procaccio V, Gygi SP, Haigis MC. 2011. Succinate dehydrogenase is a direct target of sirtuin 3 deacetylase activity. *PLoS One* **6**: e23295.

Forbes SA, Beare D, Boutselakis H, Bamford S, Bindal N, Tate J, Cole CG, Ward S, Dawson E, Ponting L et al. 2017. COSMIC: somatic cancer genetics at high-resolution. *Nucleic Acids Res* **45**: D777-D783.

Harbauer AB, Zahedi RP, Sickmann A, Pfanner N, Meisinger C. 2014. The protein import machinery of mitochondria-a regulatory hub in metabolism, stress, and disease. *Cell Metab* **19**: 357-372.

Huang LS, Sun G, Cobessi D, Wang AC, Shen JT, Tung EY, Anderson VE, Berry EA. 2006. 3-nitropropionic acid is a suicide inhibitor of mitochondrial respiration that, upon oxidation by complex II, forms a covalent adduct with a catalytic base arginine in the active site of the enzyme. *J Biol Chem* **281**: 5965-5972.

Inaoka DK, Shiba T, Sato D, Balogun EO, Sasaki T, Nagahama M, Oda M, Matsuoka S, Ohmori J, Honma T et al. 2015. Structural Insights into the Molecular Design of Flutolanil Derivatives Targeted for Fumarate Respiration of Parasite Mitochondria. *Int J Mol Sci* **16**: 15287-15308.

Jonsson T, Atwal JK, Steinberg S, Snaedal J, Jonsson PV, Bjornsson S, Stefansson H, Sulem P, Gudbjartsson D, Maloney J et al. 2012. A mutation in APP protects against Alzheimer's disease and age-related cognitive decline. *Nature* **488**: 96-99.

Kim HJ, Jeong MY, Na U, Winge DR. 2012. Flavinylation and assembly of succinate dehydrogenase are dependent on the C-terminal tail of the flavoprotein subunit. *J Biol Chem* **287**: 40670-40679.

Naamati A, Regev-Rudzki N, Galperin S, Lill R, Pines O. 2009. Dual targeting of Nfs1 and discovery of its novel processing enzyme, Icp55. *J Biol Chem* **284**: 30200-30208.

Poveda-Huertes D, Mulica P, Vogtle FN. 2017. The versatility of the mitochondrial presequence processing machinery: cleavage, quality control and turnover. *Cell Tissue Res* **367**: 73-81.

Ragin C, Blackman E, Roberts R, Butler R, Gathere S, Halliday D, Ashing K. 2017. Cancer in populations of African Ancestry: studies of the African Caribbean Cancer Consortium. *Cancer Causes Control* **28**: 1173-1176.

Robinson KM, Lemire BD. 1996. Covalent attachment of FAD to the yeast succinate dehydrogenase flavoprotein requires import into mitochondria, presequence removal, and folding. *J Biol Chem* **271**: 4055-4060.

Rotimi CN, Bentley AR, Doumatey AP, Chen G, Shriner D, Adeyemo A. 2017. The genomic landscape of African populations in health and disease. *Hum Mol Genet* **26**: R225-R236.

Schleiff E, Heard TS, Weiner H. 1999. Positively charged residues, the helical conformation and the structural flexibility of the leader sequence of pALDH are important for recognition by hTom20. *FEBS Lett* **461**: 9-12.

Sciacovelli M, Guzzo G, Morello V, Frezza C, Zheng L, Nannini N, Calabrese F, Laudiero G, Esposito F, Landriscina M et al. 2013. The mitochondrial chaperone TRAP1 promotes neoplastic growth by inhibiting succinate dehydrogenase. *Cell Metab* **17**: 988-999.

Singh R, Jamdar SN, Goyal VD, Kumar A, Ghosh B, Makde RD. 2017. Structure of the human aminopeptidase XPNPEP3 and comparison of its in vitro activity with Icp55 orthologs: Insights into diverse cellular processes. *J Biol Chem* **292**: 10035-10047.

Vaca Jacome AS, Rabilloud T, Schaeffer-Reiss C, Rompais M, Ayoub D, Lane L, Bairoch A, Van Dorsselaer A, Carapito C. 2015. N-terminome analysis of the human mitochondrial proteome. *Proteomics* **15**: 2519-2524.

Voos W, Jaworek W, Wilkening A, Bruderek M. 2016. Protein quality control at the mitochondrion. *Essays Biochem* **60**: 213-225.

Yang S, Lincoln SE, Kobayashi Y, Nykamp K, Nussbaum RL, Topper S. 2017. Sources of discordance among germ-line variant classifications in ClinVar. *Genet Med* **19**: 1118-1126.

Zhang J, Baran J, Cros A, Guberman JM, Haider S, Hsu J, Liang Y, Rivkin E, Wang J, Whitty B et al. 2011. International Cancer Genome Consortium Data Portal--a one-stop shop for cancer genomics data. *Database (Oxford)* **2011**: bar026.

Zhang Y, Deng H, Zhao Q, Li SJ. 2013. Interaction of presequence peptides with human translocase of inner membrane of mitochondria Tim23. *Biochem Biophys Res Commun* **437**: 292-299.

**Supplementary Figure Legends.**

**Supplementary Figure 1.** **SDHB expression in neoplastic and non-neoplastic cells.** Images represent SDHB expression as assessed by immunohistochemistry in **A**) junction of ccRCC tumor and normal adjacent renal tissue in proband (indicated by green dotted line), **B**) normal kidney, containing both epithelial cell-lined tubules and endothelial cells, from proband, **C)** ccRCC from an SDHA-wt patient (positive control for maintenance of SDHB expression); and **D**) GIST from an *SDHA*-mutant patient (negative control for SDHB expression). Red arrow indicates positive SDHB staining in non-neoplastic cells such as renal epitherluim and endothelial cells, and black arrow indicates SDHB-stained ccRCC tumor cells. Scale bar – 40 µm. Magnification: 40x.

**Supplementary Figure 2. Frequency of somatic mutations in *SDHx* genes**. Somatic mutation frequencies in *SDHx* genes (*SDHA*, *SDHB*, *SDHC* and *SDHD*) from over 2200 cases represented in TCGA (Cerami et al. 2012) (www.cbioportal.org), COSMIC (Forbes et al. 2017) (http://cancer.sanger.ac.uk/cosmic) and International Cancer Gene Consortium (Zhang et al. 2011) (ICGC, <http://icgc.org/)>, corrected by gene length (also see Suppl. file 5).

**Supplementary Data Files**

**Supplementary File 1. Annovar-InterVar report for the variants presented in Table 1 and original exome data report for the variants found in each family member and for all entries for SDHA.**

**Supplementary File 2. Sanger validation for the selected variants identified by exome sequencing.**

**Supplementary File 3. Maternal *SDHA* variants and their representation in ExAc and gnomAD in total and African-American Population.**

**Supplementary File 4. Sanger sequencing analysis of SDHA rs140736646 (Ala45Thr) from proband blood and tumor.**

**Supplementary File 5. Analysis of reported alterations in *SDHx* genes (*SDHA*, *SDHB*, *SDHC*, and *SDHD*).**

**Supplementary File 6. 5’UTR TGFB2 variants.**

**Supplementary File 7. Quality control data for exome sequencing experiments.**
